# Impact of Disorder, Porosity, and Surface Chemistry of Salt Templated Carbons on Capacitance

**DOI:** 10.1002/advs.202505032

**Published:** 2025-05-30

**Authors:** Amelia Klimek, Bénédicte Réty, Camélia Matei Ghimbeu, Elzbieta Frackowiak

**Affiliations:** ^1^ Poznan University of Technology Institute of Chemistry and Technical Electrochemistry Berdychowo 4 Poznan 60‐965 Poland; ^2^ Université de Haute‐Alsace Institut de Science des Matériaux de Mulhouse Mulhouse F‐68100 France; ^3^ Université de Strasbourg Strasbourg F‐67081 France; ^4^ Réseau sur le Stockage Electrochimique de l'Energie (RS2E) Amiens Cedex 80039 France

**Keywords:** active surface area, disordered carbons, electrical double‐layer capacitors, microporosity, salt‐templated carbons

## Abstract

The necessity of tailoring the structure/texture of carbons to improve the performance of aqueous‐based electrical double‐layer capacitors (EDLCs) is emphasized. A green soft‐salt templating approach allowed the preparation of a series of porous carbons for this target. The EDLCs operating in 1M Li_2_SO_4_ demonstrated a maximum capacitance of 244 F g^−1^ at 1.6 V (CsCl/KCl‐T), long‐term cycle life (288 h for LiCl/KCl‐T), and a specific energy exceeding 10 Wh kg^−1^. The physicochemical properties of carbons have been correlated with capacitance, retention, and stability. The investigation by Raman spectroscopy revealed that carbons with the increased disorder, thus, higher I_D_/I_G_ ratio, are in accord with enhanced capacitance. Active surface area (ASA) values, related to carbon defects, perfectly supported the Raman findings. Surface functionality, i.e., the phenol/ether and carboxyl groups are found to affect capacitance. The carbons showed a predominance of micropores, with a specific surface area (SSA) ranging from 2640 to 1453 m^2^ g^−1^. In sum, I_D_/I_G_, SSA, ASA, and volume of micropores are in linear proportion with capacitance at various regimes. However, the most ordered and less porous materials provided better lifespan performance. Therefore, a good compromise is required to satisfy both high capacitance and the long cycle life of EDLCs.

## Graphical abstract

Soft‐salt templating method allowed to obtain a series of tailored porous carbons for EDLCs. Neutral aqueous electrolytes enabled to reach 1.6 V. Linear dependence of I_D_/I_G_ ratio vs. capacitance values has been proven. Active surface area related to carbon defects supported the Raman findings. The most ordered and less porous materials provided better lifespan performance. Decoupling the effect of disorder degree, specific surface area, and surface functionalities on performance remains challenging.

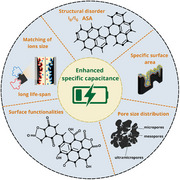

## 1 Introduction

Electrical double‐layer capacitors (EDLCs) are widely identified as high‐power energy storage devices with outstanding long‐term cycle life. Such characteristics are possible by the reversible electrostatic accumulation of ions at the electrode/electrolyte interface.^[^ ^1^ ^]^ According to the formula E = 0.5CU^2^, energy density (E) can be increased by enhancing the capacitance (C) and working voltage (U) of the EDLC. Therefore, there is a great interest in developing novel electrolytes and electrode materials that notably affect the final performance of the device.^[^ ^2^ ^]^ Ideal EDLC electrolytes should be environmentally friendly, cost‐effective, exhibit high ionic conductivity, and low viscosity, as well as offer wide stable operating voltage.^[^ ^3^, ^4^ ^]^ The most attractive neutral electrolytes, e.g., Na_2_SO_4_, Li_2_SO_4_, K_2_SO_4_, have been used for the voltage extension.^[^ ^5^, ^6^ ^]^ It was proven by *operando* studies that 1.6 V is the practical maximum operating voltage of EDLC in 1M Li_2_SO_4_, and it is possible due to the electrosorption of hydrogen in the negative electrode.^[^ ^7^ ^]^

Optimal electrode material for EDLC should be characterized by high specific surface area (SSA) and good electrical conductivity.^[^ ^8^ ^]^ Activated carbon is the most promising electrode material for EDLCs because of reasonable electrical conductivity, chemical/thermal stability, variety of morphologies, tunable porous texture, and cost‐effectiveness.^[^ ^9^, ^10^, ^11^ ^]^ In addition to the extension of the voltage which is directly linked to the used electrolyte, as well as the increase of electrode surface area, there is another option to enhance the energy density of the EDLC.^[^ ^12^ ^]^ The importance of tailoring carbon texture/structure has already been mentioned.^[^ ^13^, ^14^ ^]^ It is possible by the application of the soft‐salt templating method.^[^ ^15^ ^]^ The incorporation of a soft template creates mesoporosity, while the salt template is responsible for microporosity. Both templates are removed from the carbon structure, the soft template is thermally decomposed during the annealing and the salt template is washed out with water directly after synthesis, with the possibility of recovery for further utilization.^[^ ^16^ ^]^ This is an advantage over the traditional hard template route, where the template cannot be recovered, and its removal requires dangerous and hazardous HF acid.^[^ ^17^ ^]^ Moreover, compared to CO_2_ activation methods using biomass precursors^[^ ^18^ ^]^ (e.g., coconut shells), the soft‐salt templating methods using phenolic resin^[^ ^19^ ^]^ as a carbon source present higher carbon yield and lower gas emissions.^[^ ^20^ ^]^

*Nita et al.* ^[^ ^15^ ^]^ proposed three salt templates, i.e., KCl, NaCl, and LiCl, to obtain porous carbons. The microporosity varied with the used salt in the order KCl>NaCl>LiCl, while mesoporosity was in the opposite direction. Furthermore, the authors reported structural changes of the prepared templated carbons from more disordered (KCl) to more graphitic (NaCl, LiCl) concluded by the analysis of transmission electron microscopy (TEM) images and Raman spectra. Then, *Platek et al.* ^[^ ^21^ ^]^ advanced the works on salt‐templated carbons for EDLCs by matching the salt template cations (Li, Na, K, Rb, Cs chlorides) with the electrolyte (Li, Na, K, Rb, Cs hydroxides). It has been shown that EDLCs based on the synthesized carbons provide improved capacitance retention when the cation employed during synthesis is coupled to the cation present in the electrolyte.^[^ ^21^ ^]^

Many works focused on the correlation between capacitance and the specific surface area/pore size distribution of carbon‐based materials.^[^ ^22^, ^23^, ^24^ ^]^ In most cases, the higher the SSA, the higher the capacitance is observed. However, the lack of linearity is often noticed, especially for carbons with extraordinarily developed specific surface area, which is considered to be undesirable for EDLCs.^[^ ^25^ ^]^ Another important factor that directly affects capacitance is pore size distribution. As a rule of thumb, the closed pores do not participate in charge accumulation, and the pores smaller than the ion's size (or equal) do not participate in the formation of EDL. However, a partial desolvation of ions should not be completely excluded, particularly at strong polarization. Till now, some contrasting results have been reported on capacitance vs. pore size dependence, especially for pores ca. 1 nm. *Chmiola et al.* ^[^ ^26^, ^27^ ^]^ claimed that normalized capacitance of carbide‐derived carbon in organic electrolytes exhibits anomalous values for accessible pore sizes less than 1 nm. *Jäckel et al.* ^[^ ^28^ ^]^ revisited this issue by studies in organic and ionic liquid media considering the desolvation of ions especially under higher polarization. Yet, *B*é*guin et al.* ^[^ ^29^ ^]^ found that EDL is efficiently formed when the optimal size is assured as ≈0.7 nm for aqueous medium and ≈0.8 nm for organic medium. Interestingly, *Centeno et al.* ^[^ ^30^ ^]^ studied 28 porous carbons and found that capacitance in the organic medium is relatively constant between 0.7 and 15 nm.

Some research has already reported on the dependence of capacitance and structural ordering of the electrode material. At first, *Vix‐Guterl et al.* ^[^ ^31^ ^]^ showed that the higher the presence of structural defects in the bulk of electrode material, the higher the capacitance, in both organic and aqueous media. Moreover, *Forse et al.* ^[^ ^32^ ^]^ demonstrated that structural disordering of the carbon network, rather than pore size distribution in carbon materials, determines capacitance. Organic electrolyte (1M TEABF_4_ in ACN) and nuclear magnetic resonance technique were employed to show that the degree of disorder in the graphene‐like sheets correlates with capacitance.^[^ ^32^ ^]^ Another factor that affects the capacitance of EDLCs is the presence of surface functional groups (i.e., oxygen‐containing) on carbons.^[^ ^33^, ^34^, ^35^ ^]^

Nevertheless, there is still a lack of understanding of how the characteristics of carbon materials impact their electrochemical EDLC performance. Although some property‐capacitance correlations have been already reported, these relate to different carbons, electrolytes, and cycling conditions, which make it difficult to extrapolate a general trend. Moreover, research work, that gathers all the physicochemical properties of a series of porous carbons with electrochemical performance (capacitance, retention, and stability) in an aqueous electrolyte, is missing. In this paper, special attention was devoted to the correlation of carbon disorder, defects, porosity and surface chemistry with the capacitance of aqueous‐based EDLC. Our findings can significantly improve and optimize the design of novel materials with controlled features for sustainable energy storage systems, such as EDLCs.

## 2 Results and Discussion

### 2.1 Structural Properties of Salt Templated Carbons

The synthesis is based on the use of a phenolic‐resin which acts as a carbon source, the soft template that generates the mesoporosity, and the salt template that creates microporosity and in some cases mesoporosity, required for ion adsorption and diffusion. Moreover, at the same time, some graphitic domains are formed (depending on the cation's binding energy to the carbon core), providing good conductivity, in addition to a high specific surface area. These property modifications are possible due to the flexibility of the synthesis, involving an extensive range of synthesis parameters to be modified (e.g., pH, type and ratio of salt/soft template, temperature).^[^ ^15^ ^]^ In particular, the influence of different eutectic salt mixtures was investigated in this work. The synthesis appears advantageous due to the cost‐effectiveness and environmental friendliness of salt templates (e.g., alkali metal chlorides), which can be recovered in >80% after the synthesis (86% when the CsCl template was used) by simple washing with hot water.^[^ ^21^ ^]^ Moreover, in the case of soft‐salt templating, the C‐yield is higher (≈30%) than for conventional routes (carbonization combined with CO_2_ activation ≈25%).^[^ ^19^ ^]^

**Figure 1:**
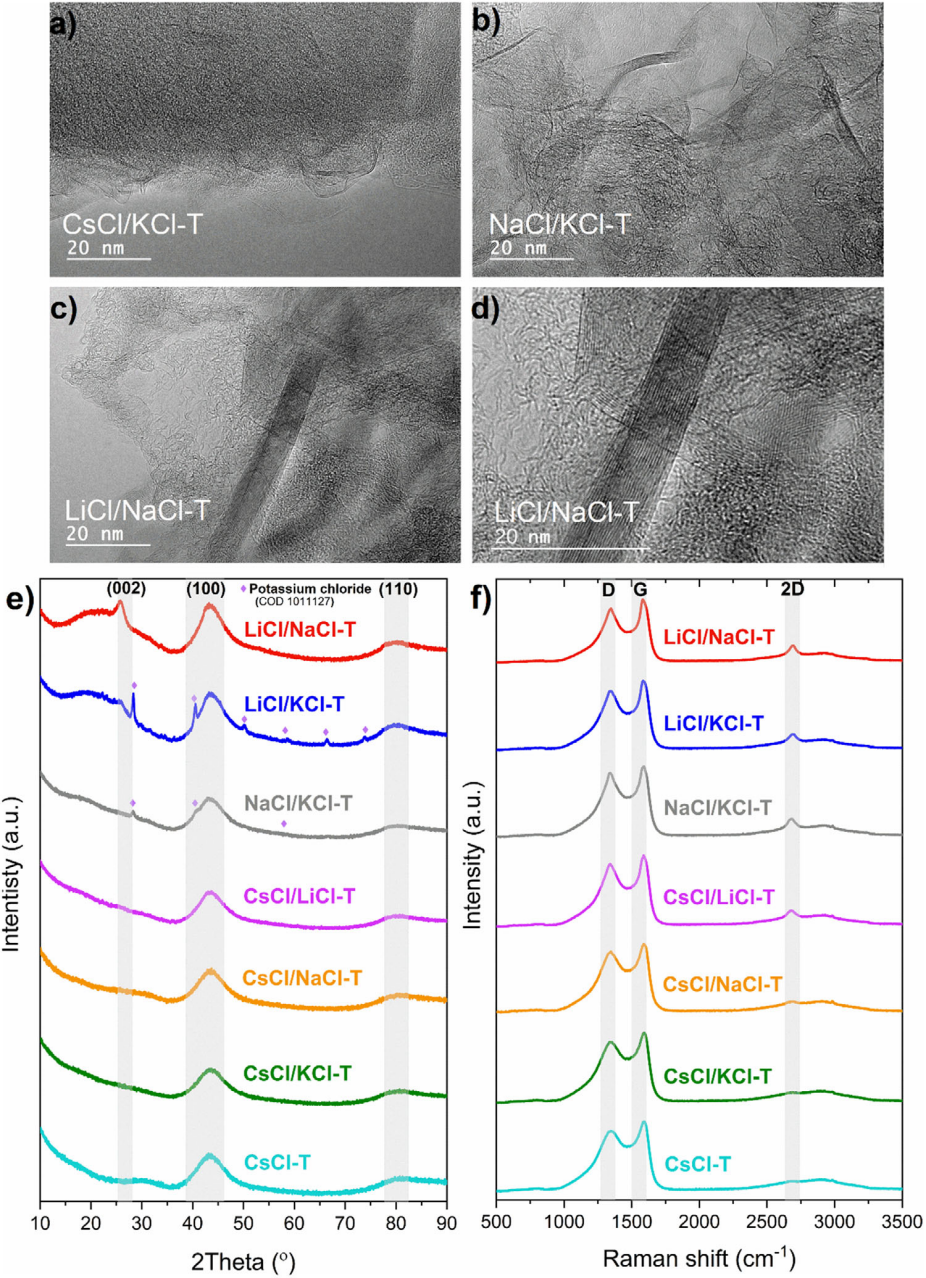
TEM images of salt templated carbons: a) CsCl/KCl‐T, b) NaCl/KCl‐T, c) LiCl/NaCl‐T, d) magnification of graphitic zone of LiCl/NaCl‐T; e) X‐ray diffraction patterns, f) Raman spectra of all salt templated carbons. T indicates template.

The local nanostructure of carbons was observed using the TEM technique, which also makes it possible to prove that the materials contain graphitic domains.^[^ ^36^ ^]^ TEM images (**Figure** **1**a–d; Figure S1a–d, Supporting Information) for a series of carbons indicate a generally disordered structure, which is beneficial for the formation of the EDL. The more disordered zones are observed mostly for carbons synthesized with cesium chloride as a salt template: CsCl‐T, CsCl/NaCl‐T, CsCl/KCl‐T (Figure S1a, b, Supporting Information; Figure 1a). The TEM images for NaCl/KCl‐T, and CsCl/LiCl‐T illustrate the existence of small, narrow graphitic domains, however, predominantly exhibit a disordered structure (Figure 1b; Figure S1c, Supporting Information). The LiCl/NaCl‐T and LiCl/KCl‐T carbons (Figure 1c; Figure S1d, Supporting Information) display extensive graphitic domains consisting of overlapping several flat graphene‐like sheets. These domains are much longer and wider (Figure 1d) than for the NaCl/KCl‐T carbon. Evolution of graphitic domains of carbon occurs above 600 °C, i.e., at temperatures above the melting point of all salts, and further develops with temperature increase.^[^ ^15^ ^]^ This process is also strongly affected by the type of salt, with LiCl providing the highest degree of graphitization due to its high cation‐π interactions with the carbon matrix (Table S2, Supporting Information).^[^ ^21^ ^]^ Therefore, the thermodynamic properties of template alkali cations affect carbon structure formation.

XRD is primarily used to characterize crystalline structures like graphite, but disordered carbons can also yield some structural information mainly because of the presence of randomly stacked graphene layers.^[^ ^37^ ^]^ XRD patterns for salt‐templated carbons (Figure 1e) show poorly defined diffraction peaks at 2 theta values of 26°, 43°, and 78°, which are associated with the hexagonal graphite (Crystallography Open Database (COD) ‐ 9011577) diffraction planes (002), (100), and (110), respectively.^[^ ^21^ ^]^ For most materials, the (002) peak is hardly visible or very broad, which aligns well with the results for other disordered‐like carbon materials.^[^ ^38^ ^]^ A small, sharp peak at ≈26° (002) suggests the presence of a graphite phase which is noticeable mainly on LiCl/NaCl‐T, in accordance with TEM images, showing extensive graphitized regions.

Furthermore, for all carbons, it is evident that the (100) peak, which is linked with the graphene lateral size (length), is definitely more intense than the (002) peak, and that intensity varies in the manner described below: LiCl/KCl‐T carbon is followed by LiCl/NaCl‐T, NaCl/KCl‐T, CsCl/LiCl‐T, CsCl/KCl‐T, CsCl/NaCl‐T, CsCl‐T. The order is related to the cation binding energy toward carbon rings.

Additionally, in the case of NaCl/KCl‐T and LiCl/KCl‐T carbons, there are visible diffraction peaks corresponding to the potassium chloride (COD 1011127) in the carbon structure. Some small quantities of KCl could not be washed out easily due to very strong carbon/alkali ion interactions, which also generate graphitic domains. Moreover, potassium as a cation is more easily incorporated than sodium because of its weaker solvation energy, which favors more interaction of potassium with the aromatic moieties.^[^ ^21^ ^]^ It is also worth mentioning that herein HCl was not used (which would have removed all the KCl) as shown in other works, but only hot water, in order to limit environmental problems.^[^ ^39^ ^]^

A reliable and effective method for investigation of structural disorder in carbon‐based materials is Raman spectroscopy; consequently, it supplies crucial additional insights to TEM and XRD studies. Raman spectroscopy involves an incident laser beam that only interacts with the π electrons of sp^2^‐hybridised carbon networks. Thus, the acquired Raman spectrum does not provide direct information concerning sp^3^‐hybridised carbon atoms.^[^ ^40^ ^]^ Generally, two main bands are observed in Raman spectra of disordered carbons.^[^ ^40^ ^]^ The first‐order graphite band (G) located at 1580–1590 cm^−1^ corresponds to the in‐plane vibrations of sp^2^ hybridised carbon atoms, rather than those in hexagonal rings. The disorder‐induced band (D) placed at 1330–1350 cm^−1^ is linked to ring breathing modes activated mainly in the presence of disorder; thus, it is directly correlated with the existence of six fold aromatic rings and exhibit intensity for materials with structural defects.^[^ ^40^, ^41^, ^42^ ^]^ It is important to note that the peak intensity changes from zone to zone, as supplied in the Raman mapping of 11 different points over the salt‐templated carbons (Figure S2a–g, Supporting Information), which indicated that the local structure is heterogeneous, especially for materials containing a mixture of disordered and graphitic domains. The average Raman spectra for all carbons (Figure 1f) show that wider D bands are associated with the utilization of the cesium chloride template, while more narrow D bands are linked to the lithium chloride template. Additionally, an overtone of the D band (called as well as the 2D band or second‐order G’ band) located at 2500–2800 cm^−1^ is related to ordered materials.^[^ ^43^ ^]^ The 2D band is mainly observed for CsCl/LiCl‐T, NaCl/KCl‐T, LiCl/NaCl‐T, and LiCl/KCl‐T carbons. In conclusion, the salt‐templated carbon structure integrates both disordered and graphitic domains (as evidenced by TEM images and XRD); thus, a well‐defined D‐band correlates with the larger presence of structural defects, while the graphitic domains are associated with an intense G‐band and the presence of 2D band. The ratio of integrated areas or intensities of disordered and ordered bands (I_D_/I_G_) is widely used to evaluate the degree of disorder.^[^ ^44^ ^]^ However, it is essential to note that, when calculating the I_D_/I_G_ ratio of disordered carbons correctly, the integrated area of the D and G bands provides a more accurate result than their intensity.^[^ ^45^, ^46^, ^47^ ^]^ Following the D and G band intensities (i.e., the heights of D and G peaks) can give misleading information for porous carbons which present a disordered structure.^[^ ^21^, ^45^ ^]^ *Sadezky et al.* ^[^ ^42^ ^]^ used the peak areas for reliable calculation of I_D_/I_G_ of different industrial carbon black materials. The pioneering work of *Ferrari et al.* ^[^ ^40^ ^]^ mentioned that in the case of amorphous/disordered carbon, it is misleading to use only the I_D_/I_G_ ratio calculated based on the peak intensities (i.e., heights). The widening of the D peak is associated with a distribution of clusters that have different orders and dimensions. Hence, the information on the less distorted aromatic rings (six rings) is reflected in the maximum intensity rather than the width, which is contingent upon the disorder. Ring orders other than six, typically diminish the peak height and increase its width. Therefore, when dealing with disordered materials, it is important to consider the peak width as well as its area. Additionally, the procedure of the deconvolution of the peaks might affect the I_D_/I_G_ ratio.

First, spectral parameters of salt‐templated carbons were determined based on the two peaks deconvolution utilizing the Lorentzian fitting function (Figure S3a, b, Supporting Information), and the values are summarized in Table S3 (Supporting Information). The I_D_/I_G_ ratios determined by the integrated area of D and G bands indicate that among all salt‐templated carbons, a clear trend is observed; the LiCl/NaCl‐T carbon has the most ordered structure with the lowest I_D_/I_G_ area (2.01), while CsCl‐T carbon presents the most disordered structure (I_D_/I_G_ area = 2.41). A similar trend is observed when compared with I_D_/I_G_ calculated from the intensities of D and G bands.

The full width at half maximum (FWHM) of the D band facilitates as well the qualitative analysis of the structural disorder of the carbon materials.^[^ ^40^ ^]^ The FWHM of the D band supports the tendency of the I_D_/I_G_ ratios, with a wider D band associated with the most disordered salt‐templated carbon (CsCl‐T). The two peaks deconvolution is very facile to realize and at the same time provides meaningful spectral information.

**Figure 2:**
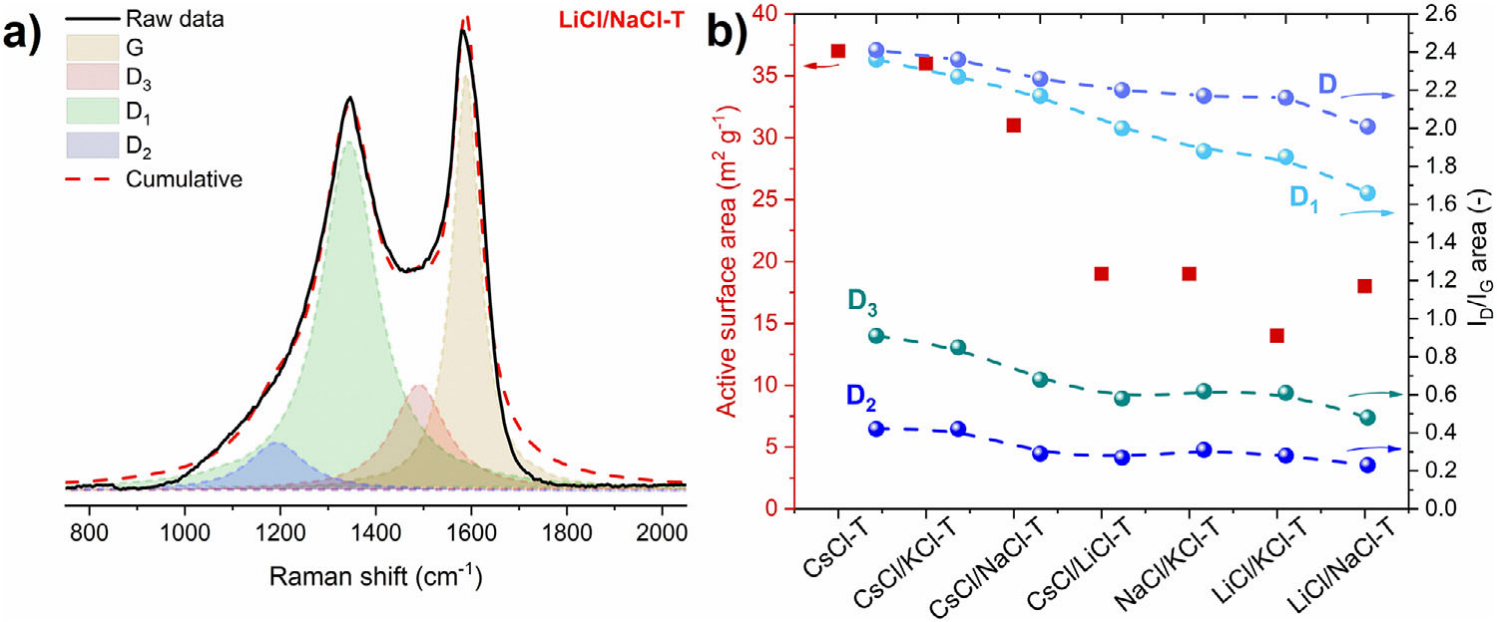
a) Raman spectrum of the most graphitic‐like salt templated carbon LiCl/NaCl deconvoluted into four peaks using Lorentzian fitting function, b) graphical representation of the dependence of active surface area and the degree of disorder (I_D_/I_G_ area (two peaks fitting), I_D1_/I_G_ area, I_D2_/I_G_ area, I_D3_/I_G_ area (four peaks fitting)) for salt templated carbons.

However, some authors have reported that the implementation of the four‐peak analysis yields a piece of more precise information than the two‐peak analysis for carbons exhibiting different levels of disorder.^[^ ^42^, ^48^ ^]^ Therefore, a four‐peak deconvolution using the Lorentzian fitting function was also performed (Figure S3c,d, Supporting Information) and spectral parameters are gathered in Table S4 (Supporting Information). Interestingly, more insightful details related to the structural defects (D_1_ band) can be concluded, associated with the presence of additional D_2_ and D_3_ bands after the deconvolution. The D_2_ and D_3_ bands are rather challenging to observe as they are inside the D and G regions, and for highly defective materials, the D and G bands exhibit broadness, resulting in the D_2_ band visibility only as a shoulder in the overall spectrum.^[^ ^49^ ^]^ *Sadezky et al.* ^[^ ^42^ ^]^ mentioned that the D_1_ band (≈1350 cm^−1^) displays a shoulder at ≈1180 cm^−1^, denoted as the D_2_ band, which provides evidence for the existence of sp^2^‐sp^3^ bonds or C─C and C═C stretching vibrations of polyene‐like structure. Some other authors claim that the origin of the D_2_ band comes exactly from transpolyacetylene segments at grain boundaries and surfaces^[^ ^50^ ^]^ or sp^2^‐based transpolyacetylene‐like chains at layer edges.^[^ ^51^ ^]^ According to *Merlen et al.* ^[^ ^49^ ^]^ the D_2_ band (sometimes called TPA for trans‐polyacetylene) has been applied to fit the Raman spectra of disordered materials like amorphous carbons with some local order (sp^2^ aromatic domains). The elevated intensity ≈1500 cm^−1^, between D_1_ and G bands can be indicated as the D_3_, which comes from the amorphous carbon fraction, comprising organic molecules, fragments, or functional groups.^[^ ^42^ ^]^ *Beyssac et al.* ^[^ ^52^ ^]^ reported that the D_3_ band is present only as a very wide band in poorly crystallized carbonaceous materials and attributed the band to defects outside the plane of aromatic layers like tetrahedral carbons. An example of such detailed peak deconvolution of Raman spectra for LiCl/NaCl‐T carbon is presented in **Figure** **2**a. Visually, the intensity and peak area after four peaks deconvolution (Figure S3c, d, Supporting Information) of both D_2_ and D_3_ bands vary from the highest for CsCl‐T (more disordered) carbon to the lowest for LiCl/NaCl‐T (more ordered) carbon. Moreover, four‐peak fitting is more accurate, since the cumulative curve matches better the raw data, than simple two‐peak fitting as shown in Figure S3a, b (Supporting Information). Taking into account, the fitted peak areas, the highest value of D_1_, D_2,_ and D_3_ peak areas as well as the maximum I_D1_/I_G_, I_D2_/I_G_, and I_D3_/I_G_ area ratios are noted for CsCl‐T carbon, while the lowest values of these spectral parameters correspond to LiCl/NaCl‐T carbon. FWHM of the D_1_, and D_2_ bands and the ratio of FWHM of D_1_, and D_2_ bands (respectively) related to the area of the G band support the tendency observed for spectral parameters mentioned already. A slightly weaker tendency is visible for the FWHM of the D_2_ band and the FWHM of the D_3_ band related to the area of the G band. The spectral parameters after four peaks of deconvolution are summarized in Tables S4 and S5 (Supporting Information). Despite several advantages of Raman spectroscopy, comprehensive information can be obtained from temperature programmed desorption coupled with mass spectrometry (TPD‐MS) and active surface area (ASA) estimation.

TPD‐MS can successfully quantify the extent of defects and active sites by quantifying the active surface area, ASA.^[^ ^53^ ^]^ The ASA is associated with various defects found not necessarily only at the carbon edges, including stacking faults, single/multiple vacancies, and dislocations. The existence of these active sites is crucial, as they have the ability to engage with other species in the electrolyte, and even improve the capacitance.^[^ ^31^ ^]^ The ASA is established through the chemisorption of O_2_ on a salt‐templated carbon which is exempt from functional groups (removed during the 1^st^ heating step of TPD‐MS), followed by the quantification of the oxygenated complexes that are formed. The evaluation of this parameter requires a particular equipment configuration and calculation method that is relatively rarely described in the existing literature,^[^ ^54^ ^]^ with only a limited number of studies documenting such values. In particular, most studies were done on graphitic or more ordered hard carbon materials,^[^ ^55^ ^]^ and information on activated carbons is missing. Only recently a protocol has been developed for accurate determination of ASA for porous activated carbons.^[^ ^56^ ^]^ The reported active surface area values vary from 37 m^2^ g^−1^ for CsCl‐T to 18 m^2^ g^−1^ for LiCl/NaCl‐T, as summarized in **Table** **1**. Figure 2b illustrates the correlation of active surface area vs. I_D_/I_G_ area ratios. Such a dependence was not reported before. It can be concluded that the highest active surface area, and the highest disorder degree of the salt‐templated carbons, highlight the contribution of the D_3_ band which is associated with the presence of functional groups. Nevertheless, O‐groups are bonded on some edge defects, while there are topological defects in the graphene basal planes as well.

**Table 1:** Textural properties determined by nitrogen adsorption (SSA, V_micro_, L_0 micro_, V_meso_, L_0 meso_, V_total_), disorder degree obtained by Raman spectroscopy (I_D1_/I_G_ area), quantities of gases released (CO_2_, CO) during TPD‐MS, and ASA of salt templated carbons.

Material	SSA [m^2^/g]	V_micro_ [cm^3^/g]	L_0 micro_ [nm]	V_meso_ [cm^3^/g]	L_0 meso_ [nm]	V_total_ [cm^3^/g]	I_D1_/I_G_ [‐]	CO_2_ [mmol/g]	CO [mmol/g]	ASA [m^2^/g]
CsCl‐T	2640	0.97	0.91	0.16	4.49	1.13	2.36	0.78	1.30	37
CsCl/KCl‐T	2446	0.92	0.86	0.15	4.70	1.07	2.27	0.73	1.37	36
CsCl/NaCl‐T	2456	0.92	0.87	0.15	5.27	1.07	2.17	0.74	1.40	31
CsCl/LiCl‐T	2202	0.83	0.85	0.12	4.52	0.95	2.00	0.62	1.06	19
NaCl/KCl‐T	1949	0.72	0.77	0.24	5.89	0.96	1.88	0.64	1.03	19
LiCl/KCl‐T	1600	0.61	0.73	0.10	5.57	0.71	1.85	0.58	1.05	14
LiCl/NaCl‐T	1453	0.52	0.75	0.42	8.45	0.94	1.66	0.77	1.26	18

### 2.2 Carbon Surface Chemistry

**Figure 3:**
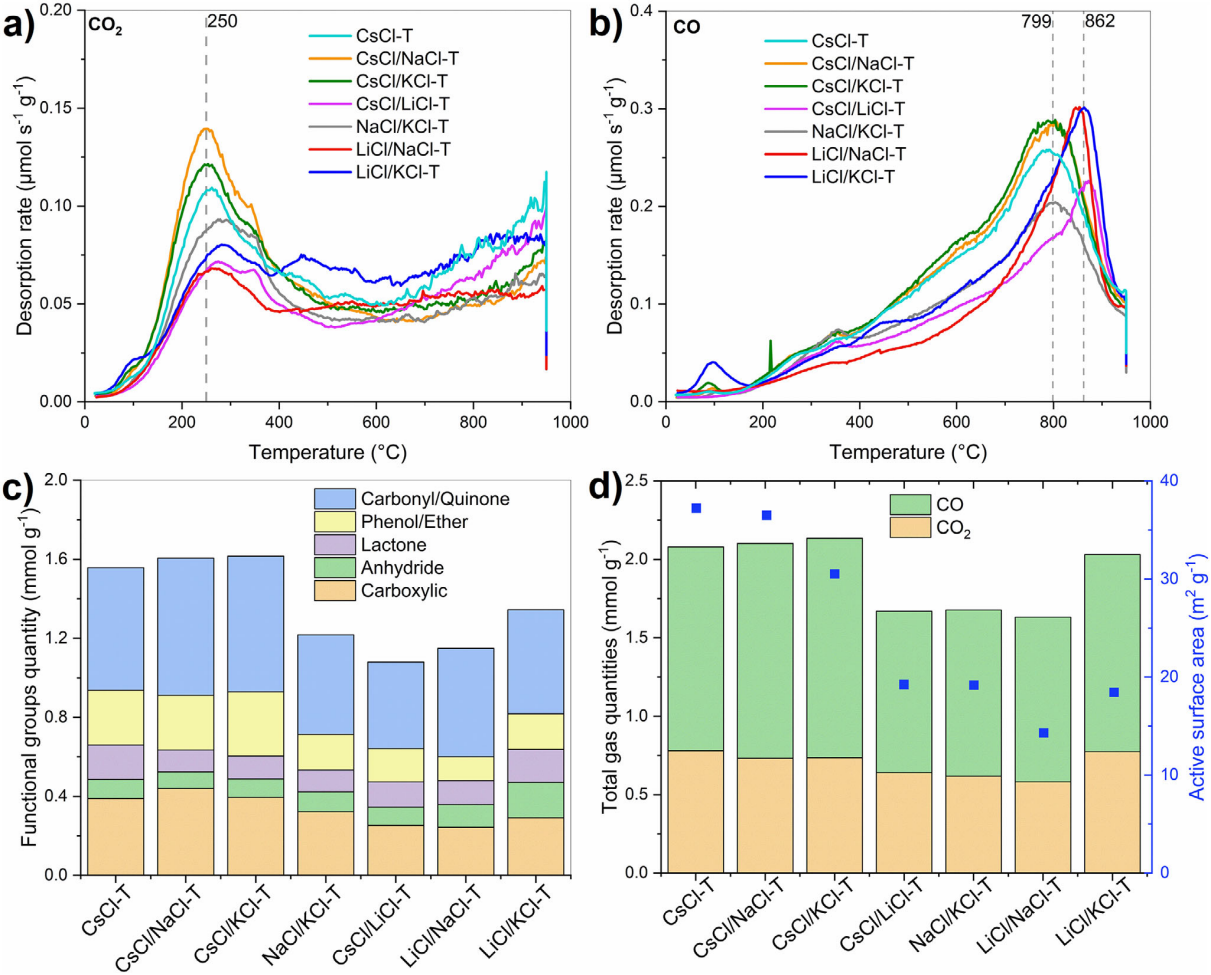
TPD‐MS gas desorption profiles of salt‐templated carbon materials for a) CO_2_, b) CO. Amounts of c) oxygen functional groups released determined by the deconvolution of the TPD‐MS peaks area, d) total gas quantities (CO, CO_2_) and active surface area for salt templated carbon materials.

To further understand the material surface chemistry, TPD‐MS was used to determine the oxygen functional groups that exist within the salt‐templated carbon structure.^[^ ^57^ ^]^ TPD‐MS is often a more judicious choice over X‐ray photoelectron spectroscopy (XPS) for analyzing carbon materials. It offers the possibility to analyze the surface chemistry in the bulk of carbon materials (in contrast to XPS, where only a top few nanometers of the material are analyzed). TPD‐MS is performed by heating to 900°C the materials, and the O‐functional groups undergo decomposition, resulting in the release of CO, CO_2_, H_2_O, and H_2_ at the temperatures that align with their thermal stability (**Figure** **3**a,b; Figure S4, Supporting Information). The main released gases during heating up the salt‐templated carbons are CO_2_ and CO. The quantity of functional groups when examining salt‐templated carbons is also of significant interest. In general, CO_2_ is emitted due to the presence of mainly acidic groups, specifically carboxyl, anhydride, and/or lactones, whereas CO is obtained from the decomposition of groups such as phenol, ether, carbonyl, and quinone.^[^ ^58^, ^59^ ^]^ One main peak is visible in the CO_2_ desorption curve (Figure 3a) for all carbons in the temperature range of 150–400°C with a maximum intensity at 250°C. The occurrence of CO_2_ groups primarily originates from the breakdown of carboxyl groups at 250°C, however, within the temperature rise, the CO_2_ peak exhibits shoulder ≈550°C, which is more pronounced for LiCl/KCl‐T, and CsCl‐T and results from carboxylic anhydrides and lactones, according to the deconvolution presented in the Figure S5 (Supporting Information). The CO desorption rate profiles exhibit one principal peak, with a maximum at 799°C for CsCl‐T, CsCl/NaCl‐T, CsCl/KCl‐T, NaCl/KCl‐T and with a maximum at 862°C for CsCl/LiCl‐T, LiCl/NaCl‐T, LiCl/KCl‐T (Figure 3b). This CO peak can be deconvoluted and quantified into more stable oxygen groups (Figure S5, Supporting Information), which occur at high temperatures such as carbonyl/quinone (800–850°C), phenol/ether (400–800°C), and anhydride (500–800°C). It can be noted that this shift of the CO peak for the latter materials is accompanied by a change in its shape, which becomes narrower, indicating the presence of more stable O‐groups, such as carbonyl/quinone. The total quantities of released gases during TPD and the exact amounts of oxygen functional groups were determined by the integration and deconvolution of the TPD‐MS peak area, respectively (Figures S4 and S5a–d, Supporting Information), and presented as a column plot for better visibility (Figure 3c). For all materials, carbonyl/quinone is the most predominant group, followed by carboxyl and phenol/ether. Anhydride and lactone are present in lesser amounts. Therefore, the basic groups are more abundant than acidic groups on the surface of the materials, while (NaCl/KCl‐T, CsCl/LiCl‐T, LiCl/NaCl‐T, and LiCl/KCl‐T) present less O‐functional groups compared to CsCl‐based materials. Interestingly, Figure 3d shows that total gas quantities (CO_2_, CO), correlates with ASA increase. A high active surface allows more O‐groups to bond on the surface, which might be beneficial for pseudo‐capacitive reactions.

Additionally, besides the desorption of CO_x_ groups, water, and hydrogen are also desorbed in small quantities (Figure S4, Supporting Information). The origin of the water is dual, comprising physiosorbed water (low temperatures) and water generated in situ by the side reactions of oxygen functional groups during heating.^[^ ^60^ ^]^ A small amount of hydrogen is released at elevated temperatures, attributed to the breaking of C─H bonds and carbon arrangements.

### 2.3 Textural Properties of Salt Templated Carbons

**Figure 4:**
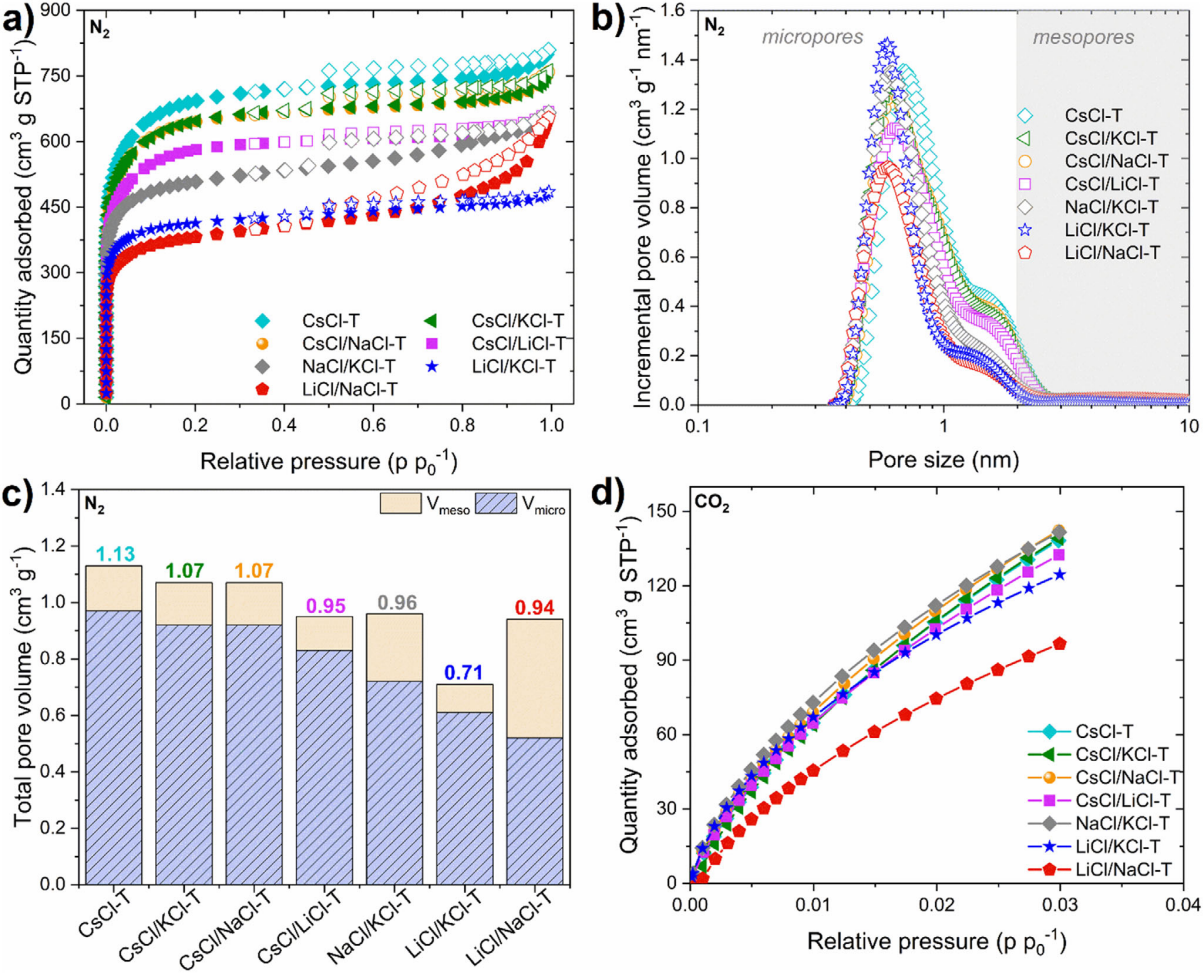
a) Nitrogen adsorption/desorption isotherms at 77K, b) pore size distribution for nitrogen adsorption using 2D‐NLDFT heterogeneous surface model from SAIEUS software, c) total pore volume and repartition of pore volumes (micro and meso) determined from nitrogen adsorption, d) carbon dioxide adsorption isotherms at 273K of salt templated carbons.

Textural properties of salt‐templated carbons were determined by the sorption of nitrogen at 77K, and the sorption of carbon dioxide at 273K. Nitrogen sorption is the most commonly used method to investigate parameters such as specific surface area and pore size distribution, which are important for the performance of EDLCs. Moreover, to achieve high power and high capacitance of EDLCs, the ion size of the electrolyte must be smaller than (or equal to) the pore size of the electrode material. Nitrogen sorption isotherms of salt‐templated carbons are shown in **Figure** **4**a, and according to the IUPAC classification, are of type I/IV, indicating mostly microporous materials with a fraction of mesopores.

The substantial increase in the quantity of adsorbed nitrogen at low relative pressures is linked to the micropores resulting from the degradation of phenolic resin (carbon precursor) and the templating effect of salt. The observed hysteresis loop at elevated relative pressures refers to the self‐organization of resin/soft‐template within the macromolecular framework and the subsequent decomposition of the template, resulting in mesoporosity creation. The formation of micelles and mesoporosity probably also was affected by the salt template, explaining the different hysteresis shapes/sizes. The values of specific surface areas are summarised in Table 1 and range from 2640 m^2^ g^−1^ for CsCl‐T to 1453 m^2^ g^−1^ for LiCl/NaCl‐T.

Figure 4b shows the pore size distribution of salt‐templated carbons, with the indication of two regions up to 2 nm (micropores) and 2–10 nm (mesopores). Micropores appear as the most interesting ones in the charge storage mechanism (EDL formation), while mesopores create pathways for electrolyte ions to reach the micropores.^[^ ^61^ ^]^ The L_0 micro_ of all carbons does not exceed 0.91 nm (the most disordered CsCl‐T), while the lowest value 0.73 nm is reached by the more ordered LiCl/KCl‐T. The L_0 meso_ changes in the opposite direction, with the larger average size (up to 8.45 nm) for the most ordered materials and the smaller average size (up to 4.49 nm) for the more disordered materials. Figure 4c displays a column plot with the repartition of the volume of micropores and mesopores within the total pore volume. The highest micropore volume is observed for the most disordered carbon, CsCl‐T (0.97 cm^3^ g^−1^), and this volume changes only slightly when CsCl is combined with other salts, which emphasizes the strong effect of CsCl on pore formation: CsCl/KCl‐T (0.92 cm^3^ g^−1^), CsCl/NaCl‐T (0.92 cm^3^ g^−1^), and CsCl/LiCl‐T (0.83 cm^3^ g^−1^). When mixtures of Li, Na, and/or K chlorides are used, in the absence of CsCl, the micropore volume decreases, as a result of the predominant effects of these salts for graphitization of materials: NaCl/KCl‐T (0.72 cm^3^ g^−1^), LiCl/KCl‐T (0.61 cm^3^ g^−1^), LiCl/NaCl (0.52 cm^3^ g^−1^). In addition, for the latter materials, the maximum volume of mesopores (0.42 and 0.24 cm^3^ g^−1^ for LiCl/NaCl‐T and NaCl/KCl‐T, respectively) is achieved and can be attributed to the voids between stacked graphene‐like layers. The decomposition of soft‐template and the carbon‐cation interactions resulted in the formation of such graphitic domains and mesopores. Additionally, lithium and sodium cations have the highest hydration enthalpies among all used cations; therefore, the biggest hydrated ion diameter influenced the formation of micelles and enhanced the ratio of mesopores (Table S2, Supporting Information). All parameters (SSA, V_micro_, L_0 micro_, V_meso_, L_0 meso_) based on nitrogen sorption data are summarised in Table 1. Then, for further investigation of the microporosity of salt‐templated carbons, sorption of carbon dioxide at 273K was used (Figure 4d; Table S6, Supporting Information). The CO_2_ molecule can access the ultramicropores (< 0.7 nm) due to its size and better diffusion, which is favored by the higher analysis temperature (273K) than for N_2_ sorption (77K). The volume of ultramicropores calculated with CO_2_ sorption data exhibited lower values compared to those assessed through N_2_ sorption, with the lowest volume for the most ordered carbon (LiCl/NaCl‐T) and the highest for the most disordered carbons (CsCl‐T, CsCl/KCl‐T). However, it is noteworthy that ≈50–60% of the specific surface area is placed in the ultramicropores region. It means that the pore confinement in the range from 0.3 to 0.7 nm plays an important role in the charge accumulation. The findings on the synthesized salt‐templated carbon texture indicated that alkali cation sizes affect micropore volume and average diameter – larger cation sizes correspond to increased micropore volume and average diameter of micropores, consistent with the SSA values.

### 2.4 Electrochemical Investigation of Salt Templated Carbons

**Figure 5:**
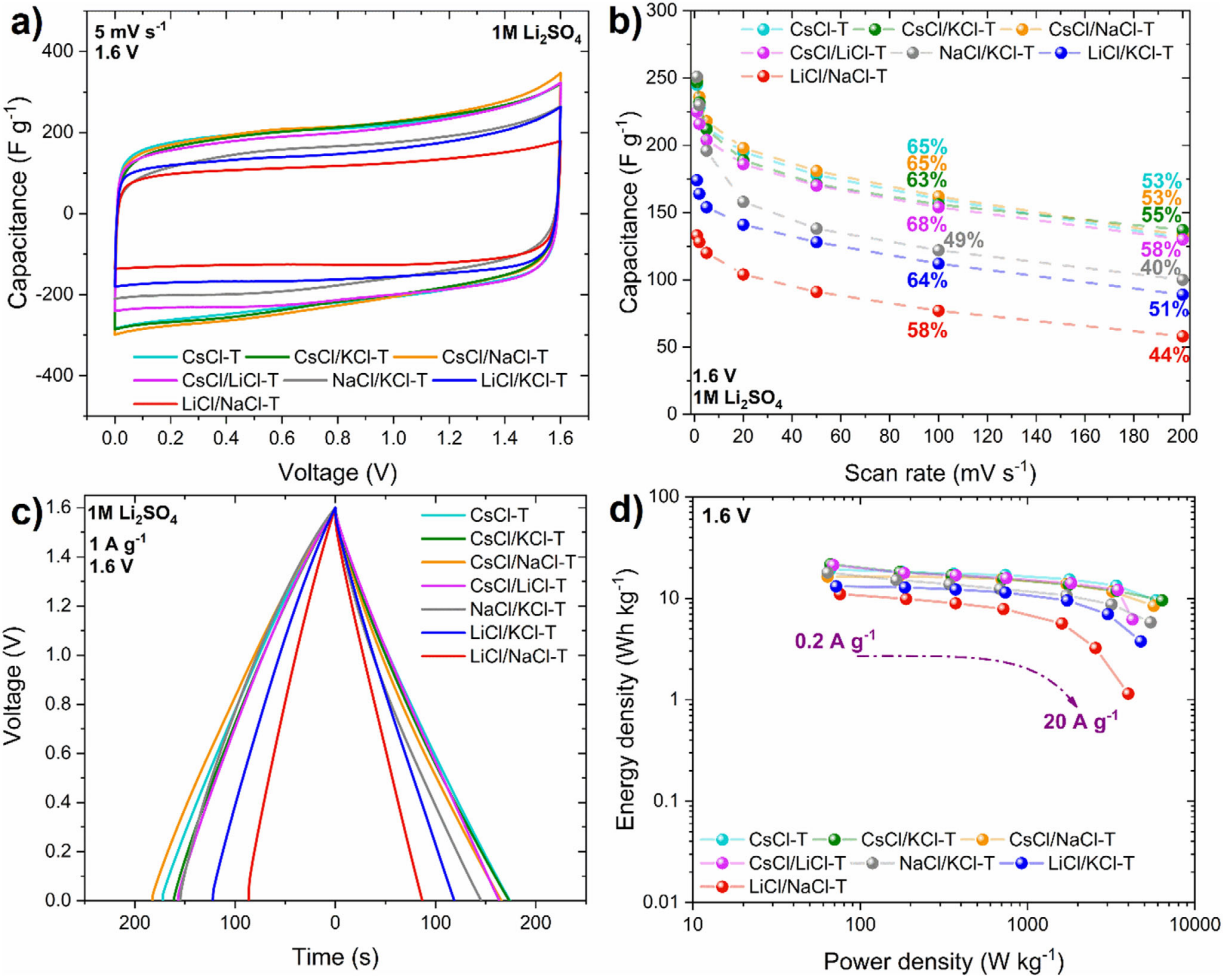
Electrochemical performance of salt‐templated carbons‐based EDLCs operating in 1M Li_2_SO_4_ at 1.6 V: a) cyclic voltammetry at 5 mV s^−1^, b) gravimetric capacitance (F g^−1^) vs. scan rate (mV s^−1^), c) galvanostatic charge/discharge at 1 A g^−1^, d) Ragone plot.

The electrochemical investigation started with the construction of two‐electrode EDLCs employing electrodes prepared from salt‐templated carbons, 1M Li_2_SO_4_ as the electrolyte, and glass fiber as a separator. The maximum stable operating voltage of the system was set as 1.6 V according to previous studies on the stability of 1M Li_2_SO_4_ as an electrolyte.^[^ ^7^, ^62^ ^]^ The voltammograms of all the systems are presented in **Figure** **5**a (5 mV s^−1^) and Figure S6a,b (Supporting Information) (100, 200 mV s^−1^). The CV curves at 5 mV s^−1^ displayed an ideal, rectangular shape, indicating pure electrostatic attraction of ions. Therefore, the charge storage mechanism is based on the formation of an electrical double layer. The characteristic shape of CV curves suggests optimal accessibility of ions to pores and adequate conductivity of the electrode materials. However, the voltammograms at elevated scanning rates, i.e., 100, 200 mV s^−1^, show a slight deviation from the typical rectangular shape with a decrease in capacitance (Figure 5b; Figure S6a,b, Supporting Information).

As demonstrated by the galvanostatic charge/discharge curves at 1 A g^−1^, all EDLCs based on salt‐templated carbons exhibited an ideal triangular shape (Figure 5c). The capacitance values calculated by integrating area under the discharge curve at 1 A g^−1^ range from 197 F g^−1^ for CsCl‐T, to 100 F g^−1^ for LiCl/NaCl‐T‐based EDLCs. Salt‐templated carbons present higher capacitance values than typically used commercially available carbons, as shown in Table S7 (Supporting Information).

The energetic efficiency during galvanostatic charging/discharging was calculated by dividing the integrated values of discharge and charge energy, then multiplying by 100 to express the final value as a percentage, following the approach outlined in ref.[63] The energetic efficiency at 1 A g^−1^ ranges from 84% for CsCl‐T to 70% for NaCl/KCl‐T‐based EDLCs. At extremely high current regime (10 A g^−1^), when the electrode material is requested for rapid charge/discharge (≈12 s), deviation from an ideal triangular shape of GCD is observed, with a decrease in initial capacitance of almost half, i.e., from 185 F g^−1^ at 0.2 A g^−1^ to 95 F g^−1^ at 20 A g^−1^ (capacitance retention 51%) for CsCl/NaCl‐T based EDLC (Figure S7a,b, Supporting Information). However, if a system works well at high current densities (> 10 A g^−1^) it indicates that such EDLC is characterized by quite good electrochemical performance with fast kinetics and good charge propagation desired for high‐power applications. The effect of the beneficial properties of salt‐templated carbons on the energy and power metrics of EDLC is evident in the Ragone plot (Figure 5d). Specific energy higher than 10 Wh kg^−1^ is observed in the wide power range.

Charge propagation of capacitor materials was examined using electrochemical impedance spectroscopy, and results are presented in Figure S7c,d (Supporting Information). The Nyquist plot (Figure S7c, Supporting Information) reveals relatively similar equivalent series resistance values, ranging from 0.49 Ω for CsCl/KCl‐T to 0.61 Ω for LiCl/KCl‐T based EDLCs. These values do not provide a trend, the resistance of the system can be impacted by the cell assembly.^[^ ^64^ ^]^ In the high‐frequency zone, the graphs exhibit a semicircle, followed by a linear segment in the low‐frequency region. Capacitance retention as a function of frequency (Figure S7d, Supporting Information) illustrates a clear plateau in the low frequencies, indicating a purely capacitive charge. Moreover, the charge capability is slightly increased in the case of EDLCs based on carbons utilizing cesium cation as a salt template, resulting in improved ion mobility inside the porosity.

Volumetric capacitance is a crucial parameter, especially from the industrial point of view, where compact and miniaturized devices including wearable electronics require reduced space. Figure S8 (Supporting Information) shows the volumetric capacitance *vs*. current density for salt‐templated carbons‐based EDLCs operating in 1M Li_2_SO_4_ up to 1.6 V.

The values range from 77 F cm^−3^ for the most porous carbons (based on the cesium salt template) to 49 F cm^−3^ for more ordered carbons (based on the lithium salt template) at 1 A g^−1^. A trend is consistent for both the gravimetric and volumetric capacitances of salt‐templated carbons. Self‐discharge is an unavoidable issue for EDLCs that refers to the loss of voltage and finally stored energy.^[^ ^65^ ^]^ This voltage drop occurs when EDLC remains at open circuit condition for 12 h after charging.^[^ ^66^ ^]^ The main causes of self‐discharge are charge redistribution in pores,^[^ ^67^ ^]^ faradaic reactions,^[^ ^68^ ^]^ and so‐called ohmic leakage.^[^ ^69^ ^]^ *Galek et al.* ^[^ ^70^ ^]^ reported that the voltage dropped from 1.6 to 0.97 V (39% loss of the voltage) for EDLC based on commercial carbon YP80F electrodes and 1M Li_2_SO_4_ as the electrolyte. In comparison, the EDLCs based on salt‐templated carbons and the same electrolyte show slightly reduced loss of the voltage ranging from 29% for LiCl/NaCl‐T to 36% for CsCl‐T, LiCl/NaCl‐T, and LiCl/KCl‐T (Figure S9, Supporting Information). When the electrochemical behavior of positive and negative electrodes of EDLCs based on selected carbons (CsCl‐T and LiCl/NaCl‐T) is studied individually at relatively low current density and scan rate, nearly square‐shaped cyclic voltammograms, and ideal triangular‐shaped galvanostatic charge/discharge curves are observed for LiCl/NaCl‐T based EDLC (Figure S10c,d, Supporting Information). It is analogous to characteristics obtained in the same electrolyte (1M Li_2_SO_4_).^[^ ^71^ ^]^ Contrarily, there is a visible deviation from ideal CV and GCD curves for CsCl‐T‐based EDLC (Figure S10a,b, Supporting Information). In the case of the most disordered carbon (CsCl‐T), the decomposition of the electrolyte (1M Li_2_SO_4_) is more significant due to the presence of a higher amount of oxygenated functionalities and a more defective structure. The decomposition of the electrolyte also takes place in LiCl/NaCl‐T‐based EDLCs, however, it is less evident due to the lower amount of surface functionalities. Also, the shape of CV is more resistive for CsCl‐T carbon‐based EDLC owing to higher disorder, i.e., less conductivity of the material. In addition to the importance of enhancing the energetic performance of EDLCs, the electrochemical stability, and long‐term performance are also recognized as critical factors. Galvanostatic cycling stands out as a widely predictable and frequently employed method for achieving that objective. Nonetheless, this technique demands a substantial amount of time. Herein, a less time‐demanding voltage holding test, commonly referred to as floating, and widely employed in the industry, is used to demonstrate the lifespan stability of EDLCs based on selected salt‐templated carbons and 1M Li_2_SO_4_ as an electrolyte.^[^ ^72^ ^]^

**Figure 6:**
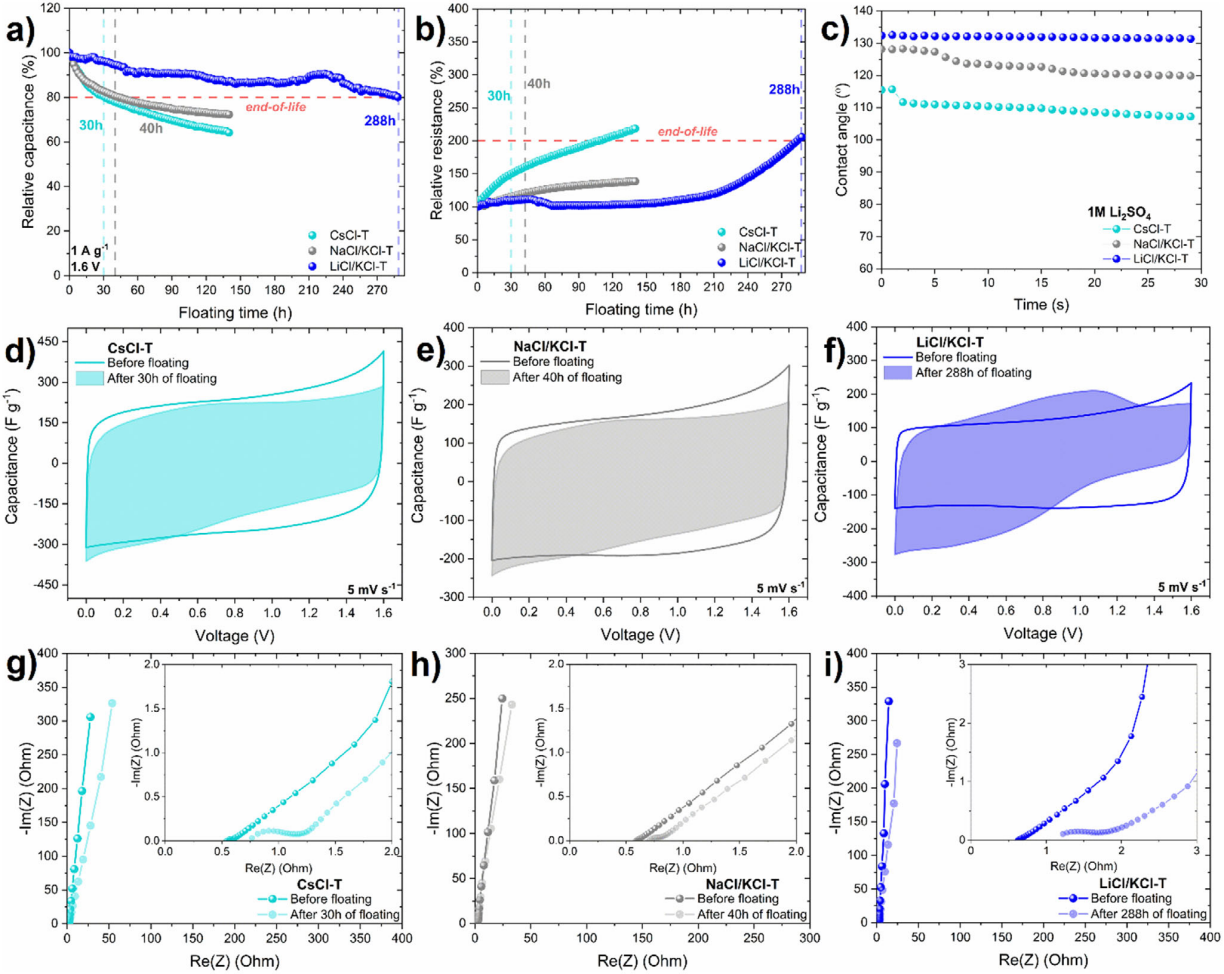
Electrochemical performance of selected salt templated carbons‐based EDLCs operating in 1M Li_2_SO_4_ at 1.6 V, 1 A g^−1^ a) relative capacitance (%) vs. floating time (h), b) relative resistance (%) vs. floating time (h), c) Contact angle measurements for selected salt templated carbon materials in 1M Li_2_SO_4_, d–f) cyclic voltammogram at 5 mV s^−1^ before and after the floating test, g‐i) Nyquist plot at 0 V before and after the floating test.

Each EDLC was charged to a maximum voltage of 1.6 V (1 A g^−1^), subsequently maintaining a voltage hold at 1.6 V for 2 h. During each voltage hold, additionally, CV and EIS were recorded. Capacitance and equivalent series resistance (ESR) values were assessed every 2 h until at least one of the end‐of‐life criteria was reached. Three carbons with differences in properties and performance were selected for long‐time performance testing, i.e., the most porous/less graphitic (the highest capacitance) CsCl‐T, the more graphitic/less porous (lower capacitance) LiCl/KCl‐T and intermediate porosity‐graphitization and performance NaCl/KCl‐T. As can be seen in **Figure** **6**a, the plot of relative capacitance vs. aging time at 1.6 V shows that a 20% decrease of initial capacitance was reached after 30 h for CsCl‐T, 40 h for NaCl/KCl‐T, and 288 h for LiCl/KCl‐T based EDLCs. The second end‐of‐life criterion, i.e., a 100% increase of relative resistance, was reached only in the case of LiCl/KCl‐T‐based EDLC, after 288 h. Other systems based on CsCl‐T and NaCl/KCl‐T reached a 150% and 120% increase in relative resistance, respectively (Figure 6b). Interestingly, from the industrial point of view, the term end‐of‐life is extended by the device application.^[^ ^73^ ^]^ End‐of‐life based on capacitance retention is more crucial in the case of prolonged pulses, while end‐of‐life caused by resistance retention is more important for brief, high‐power pulses.^[^ ^72^ ^]^ Therefore, the differences in reaching end‐of‐life criteria by the EDLCs based on salt‐templated carbons may extend the variety of possible applications.

The wettability of electrode materials by the electrolyte is crucial for efficient EDL formation, significantly impacting the performance of the device. Good wettability increases the ion‐ accessible surface area, facilitates ion diffusion within the porosity and promotes ion mobility.^[^ ^74^ ^]^ Therefore, insufficient wettability results in underutilization of surface area, which in turn leads to diminished gravimetric capacitance and retention at fast regimes. The contact angle value measured for CsCl‐T is the lowest one, i.e., 111°, indicating a more hydrophilic surface compared to 127° for NaCl/KCl‐T and 131° for LiCl/KCl‐T (Figure 6c). Indeed, the highest amount of O‐containing functional groups for CsCl‐T improves the wettability of the material and can slightly improve electrochemical performance by providing more pseudocapactiance.^[^ ^75^ ^]^

The voltammograms and galvanostatic charge/discharge curves for EDLCs before and after prolonged floating (Figure 6d–f; Figure S11a–c, Supporting Information) show a significant decrease in capacitance at voltages exceeding 0.8 V. EDLCs polarised at U_max_ = 1.6 V are susceptible to the parasitic reactions. The humps (visible especially for LiCl/KCl‐T, Figure 6f) are linked to the redox activity of O‐groups and pH variation of the electrode/electrolyte interface.^[^ ^76^ ^]^ Partial oxidation of the positive electrode may occur due to the prolonged floating time due to the carbon corrosion involving side reactions accompanied by the emission of CO and CO_2_. It results in the production of solid‐state deposits (Li_2_CO_3_) due to the reaction of electrolyte with CO_x_ by‐products of carbon corrosion, which gradually blocks the porosity at elevated voltage.^[^ ^71^, ^77^ ^]^ Figure 6g–i illustrates the impact of accelerated aging on the Nyquist plots of EDLCs based on selected carbons. The slight increase in resistance is visible for CsCl‐T (ESR +50%) and for NaCl/KCl‐T (ESR +20%)‐based EDLCs. A noticeable, two‐fold increase of resistance is obvious for the LiCl/KCl‐T‐based system, which reached two end‐of‐life criteria after the longest time (288 h). Concluding, the aging of the systems operating in such electrolytes is primarily attributed to the decline in electrode performance due to the formation of new surface functionalities,^[^ ^7^ ^]^ collapse of porosity,^[^ ^78^ ^]^ pH modification in both electrode/electrolyte interfaces^[^ ^7^ ^]^ and possible accumulation of corrosion products originating from the electrode material and/or stainless steel current collectors.^[^ ^62^ ^]^ The EDLC based on CsCl‐T salt templated carbon reached its end‐of‐life after only 30 h. The CsCl‐T is the most disordered material of all, and its enhanced porosity and O‐functionalities are susceptible to adverse structural alternations during ion transport. In contrast, the more graphitic‐like and less porous materials exhibit superior long‐term performance (LiCl/KCl‐T). Obviously, there is a trade‐off to be found between the high capacitance values and the lifespan of EDLC.

### 2.5 Correlation of Salt Templated Carbons Properties with Gravimetric Capacitance

**Figure 7:**
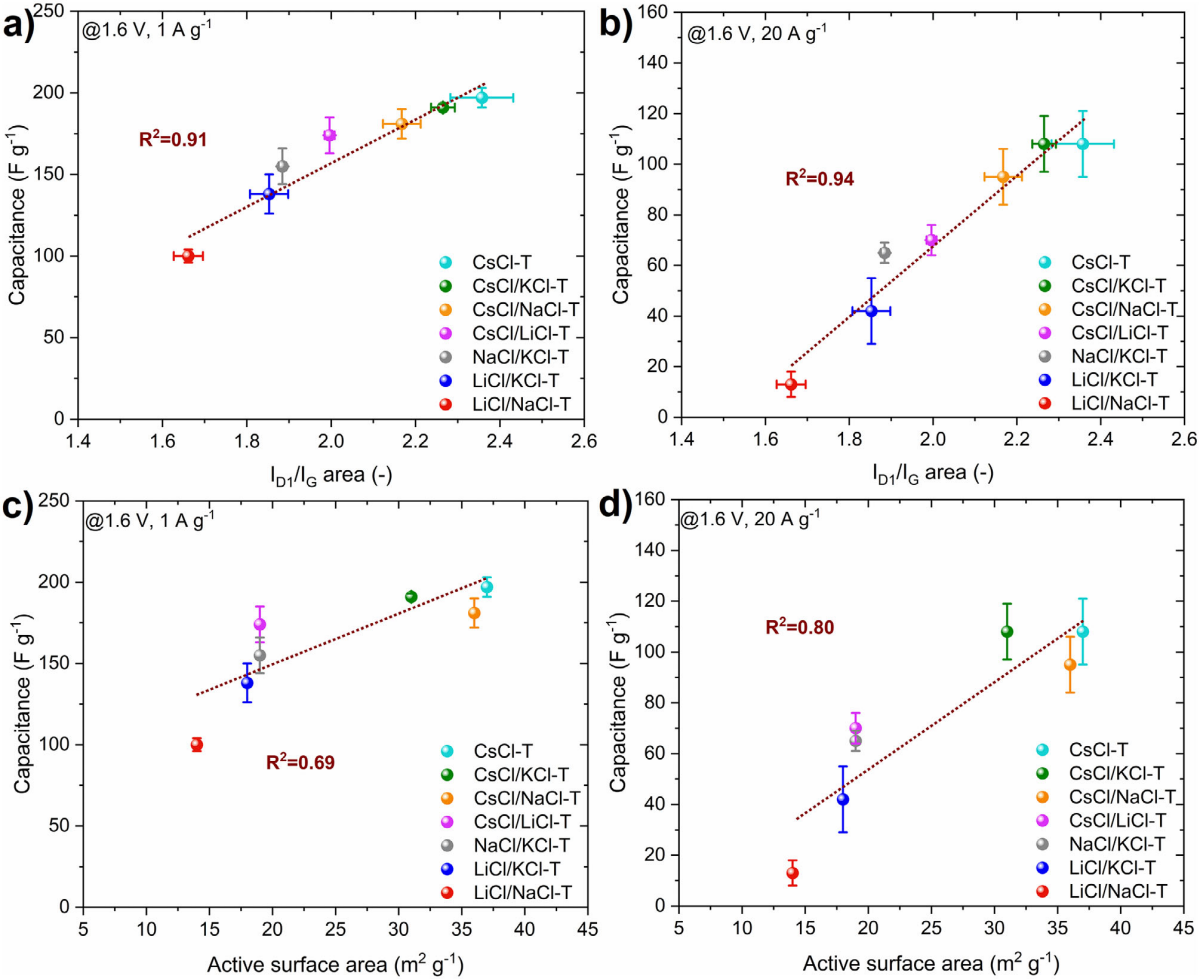
Correlation of gravimetric capacitance and I_D1_/I_G_ ratio at different current densities a) 1 A g^−1^, b) 20 A g^−1^ of salt templated carbons. Correlation of gravimetric capacitance and active surface area at different current densities c) 1 A g^−1^, d) 20 A g^−1^ of salt‐templated carbons.

In‐depth textural/structural characterization of synthesized salt templated carbons combined with the electrochemical performance of aqueous‐based EDLC, allowed significant insights to be gained. Such advanced physicochemical and electrochemical characterization by various and complementary techniques is required to prove that the correlations that were found are reliable and consistent. Moreover, many researchers, while correlating their structural and/or textural findings with gravimetric capacitance, neglect the role of the current density applied and show results in accordance with one selected regime. Figure S12a–c (Supporting Information) presents the correlations of Raman spectral parameters obtained with two peaks fitting (I_D_/I_G_, FWHM D) and gravimetric capacitance for salt‐templated‐based EDLCs operating in 1M Li_2_SO_4_. The general tendency of higher capacitance values with higher disorder of the materials is observed in each case with good coefficients of determination (R^2^). A small deviation from linearity (R^2^ = 0.82) is shown for capacitance vs. FWHM of the D band, probably caused by the slight inaccuracy of the two peaks fitting procedure. For comparison, the spectral parameters after four peaks fitting and gravimetric capacitance are shown in **Figures** **7**a, S13, and Table S8 (Supporting Information). The findings from the two peaks fitting are confirmed, i.e., the more disordered carbons (higher I_D1_/I_G_ ratio, higher FWHM D_1_) tend to have higher gravimetric capacitances. As more detailed, the spectral parameters after four peaks deconvolution by the Lorentzian fitting function are employed for further analysis and correlations with gravimetric capacitances. Figure 7a,b and Figure S14a–e (Supporting Information) depict the correlation of degree of disorder (I_D1_/I_G_ area) with the gravimetric capacitance of carbons operating in 1M Li_2_SO_4_ at different current densities (0.2 – 20 A g^−1^). Gravimetric capacitance clearly increases with the higher disorder of the salt‐templated carbons (higher I_D1_/I_G_ area ratio) as summarized in Table 1. and Table S8 (Supporting Information) with satisfactory R^2^ (e.g., 0.91 at 1 A g^−1^, 0.95 at 20 A g^−1^). It is clear that, the higher the disorder and amount of defects, the higher the capacitance. The defects in the form of topological and edge sites cause uneven distribution of delocalized electrons in the carbon matrix. Edge‐oriented sites are more favorable for the electrostatic attraction of ions.^[^ ^79^ ^]^ Additionally, in disordered carbon, the distance between the graphene domains (d_002_) is much higher than for graphitic carbons (≈0.400 nm for very disordered carbon vs. 0.335 nm for graphite).^[^ ^80^ ^]^ However, it cannot be precisely calculated in the case of salt‐templated carbons due to the absence of a well‐defined 002 XRD peak. The larger interlayer spacing in the CsCl‐T carbon (as suggested by the extremely broad and low intense 002 XRD peak) allows better ion transport, compared to the LiCl/NaCl‐T carbon. The latter presents a more intense, broad 002 XRD peak – linked to the disordered carbon with few stacked graphenes and in addition, a sharp 002 XRD peak – associated with graphite, thus smaller d_002_ distance results in more difficult ion diffusion/adsorption. As the d_002_ from XRD cannot be determined for salt‐templated carbons, the Tunistra & Koenig relation can be applied to Raman results to calculate L_a_ (the size (height) of stacked graphene layers),^[^ ^40^, ^81^ ^]^ as summarized in Table S9 (Supporting Information). It is clearly seen that L_a_ ranges from 7.98 nm for the CsCl‐T to 9.56 nm for the LiCl/NaCl‐T templated carbon. Therefore, the graphitic domains are increasing with the cation‐π binding energy, in line with the TEM results. It is expected that the crystalline structure of the LiCl/NaCl‐T enhances the electron mobility, but more ordered and higher stacked graphitic domains with reduced distance between the graphene layers are contributing to the capacitance decrease (along with SSA decrease). Hence, it is perfectly supporting our correlation of the I_D_/I_G_ ratio vs. capacitance.

In Figure 7c,d, the ASA and gravimetric capacitance correlate with R^2^ 0.69 (1 A g^−1^) and 0.80 (20 A g^−1^) which is in accordance with the Raman findings. It is worth mentioning that the correlation is the most linear at the highest current density applied (20 A g^−1^), where the activity of surface functionalities is excluded. There is a better correlation with the I_D1_/I_G_ than for the ASA vs. capacitance. To explain that behavior, the differences between Raman spectroscopy and ASA estimation must be clarified. Raman spectroscopy is a local technique that analyses ≈300 nm of the surface, contrary to the TPD‐MS (ASA estimation), where the entire material is analyzed. Moreover, Raman spectroscopy has several advantages, but it has been noted that this type of analysis cannot quantify the exact area occupied by the defects or quantify functional groups present in the carbon. The ASA is associated with various defects found both on the basal planes and at the carbon edges, including stacking faults, single/multiple vacancies, and dislocations. Nevertheless, during ASA estimation, oxygen functionalities are bonded rather on some edge defects, while there are defects in the graphene basal planes as well (monitored by Raman spectroscopy). Thus, by Raman spectroscopy and ASA, we are not analyzing exactly the same type of defects. Both ASA and Raman spectroscopy are complementary techniques, with slight differences as explained above, therefore, some variations in the correlations with electrochemical performance are possible. Interestingly, a similar dependence of gravimetric capacitance and degree of disorder was proven by NMR studies on activated carbon^[^ ^32^ ^]^ and TPD‐MS on graphitic nanocarbon.^[^ ^31^ ^]^ Moreover, the correlation of gravimetric capacitance vs. I_D1_/I_G_ area ratio and FWHM of the D_1_ band (respectively) support the recent findings of *Forse et al.* ^[^ ^48^ ^]^ The discrepancy is observed in the case of the I_D_/I_G_ ratio calculated from peak intensities in the reported work,^[^ ^48^ ^]^ which should not be considered as appropriate procedure for disordered carbons. Our investigation of I_D_/I_G_ area ratio vs. gravimetric capacitance has been conducted using an aqueous electrolyte, contrary to the findings of other groups ^[^ ^48^ ^]^ where the organic electrolyte was applied. In both cases, for aqueous and organic electrolytes, the carbon functional groups may influence the total charge of the carbon.^[^ ^82^ ^]^ Certainly, in the aqueous electrolytes, the protonation or deprotonation of certain oxygen‐containing groups (e.g., carboxylic, phenolic, carbonyl, quinone) is contingent upon local pH changes. Furthermore, the presence of oxygen surface functionalities, which are often polar, enhances the hydrophilicity of the material.^[^ ^83^ ^]^ Such affinity to water can be attributed to the hydrogen bonds formed between water molecules and the surface oxygen atoms.^[^ ^84^ ^]^ On the other hand, heteroatoms such as oxygen create active sites for the electrolyte decomposition,^[^ ^85^ ^]^ diminishing the conductivity of carbon and, in turn, charge propagation.

**Figure 8:**
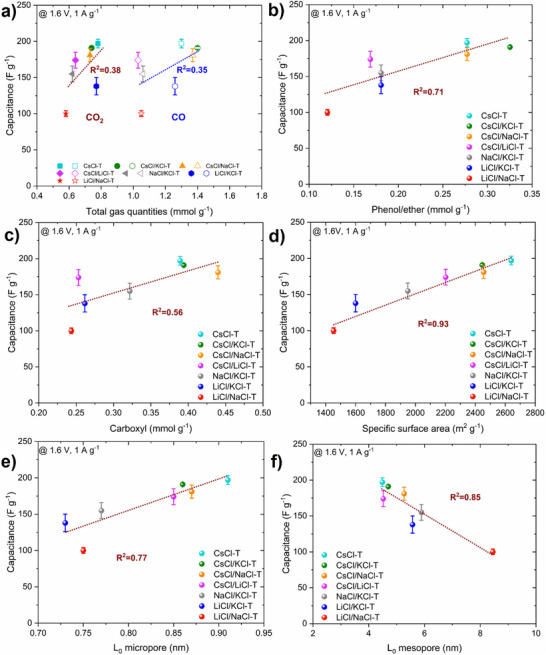
Correlation of gravimetric capacitance (1 A g^−1^, 1.6 V) and a) total CO_2_ and CO quantities, the quantity of O‐functional groups: b) phenol/ether, c) carboxyl, d) specific surface area determined from nitrogen sorption at 77K, e) the average diameter of micropore, f) the average diameter of mesopore of salt templated carbons.

Ultimately, it is important to admit that numerous additional variables may influence capacitance, other than simply the local ordering of carbons. Therefore, elemental analysis was used to estimate the % of oxygen, and TPD‐MS was used to analyze and quantify the oxygen functionalities that impact the performance of EDLCs. The oxygen content in salt‐templated carbons verified by elemental analysis ranges from 2.1% (LiCl/KCl‐T) to 3.5% (CsCl/NaCl‐T), which can be considered as small amounts (Table S10, Supporting Information). Owing to the careful analysis of TPD‐MS results, the amount of CO and CO_2_ released by the surface group materials can be correlated with the gravimetric capacitance (**Figure** **8**a). Linear dependence is observed; with a higher quantity of released gases, the gravimetric capacitance is increasing. Elevated amounts of CO and CO_2_ are associated with more disordered salt‐templated carbons and are linking other findings from Raman spectroscopy and active surface area studies. It can be seen that the R^2^ is small (< 0.4). This may be related to the presence of several functional groups on the carbon surface, which have different interactions with the electrolyte, impacting the capacitance in diverse ways.

Indeed, if the exact quantities of surface functionalities determined after deconvolution of TPD‐MS peaks are correlated with the gravimetric capacitance at 1 A g^−1^ (Figure 8b,c), good linear dependence is observed for gravimetric capacitance vs. quantity of phenol/ether and carboxylic functional groups, with higher R^2^ (0.71 and 0.56, respectively), while lack of linearity is observed for carbonyl/quinone, anhydride, and lactone functional groups.

Moreover, it is important to mention that the possible redox activity of surface functional groups is excluded at elevated current densities (i.e., 20 A g^−1^) due to the insufficient time for redox reactions, therefore, such correlations are slightly inconsistent with gravimetric capacitances recorded at 1 A g^−1^. Another factor is that not all the defects have a favorable effecton capacitance improvement. In the case of salt‐templated carbons, a better correlation of ASA coming from the contribution of phenol/ether and carboxyl groups vs. capacitance was found in our previous work.^[^ ^56^ ^]^ Contrary, carbonyl/quinone, anhydride, and lactone‐derived ASA did not show high R^2^. The specific surface area and pore size distribution (total volume and average diameter) of the materials are the other factors that influence the gravimetric capacitance as well as the degree of disorder. Figure 8d and Figure S15a (Supporting Information) illustrate the correlation for specific surface area (N_2_ sorption at 77K) of salt‐templated carbons and gravimetric capacitance of EDLC based on these carbons at 1 and 20 A g^−1^, respectively. It is evident that gravimetric capacitance rises with the increase of specific surface area, presenting a high coefficient of determination factors, i.e., R^2^ = 0.93 for 1 A g^−1^ and R^2^ = 0.95 for 20 A g^−1^ current densities. The EDL develops at the carbon/electrolyte interface, according to the formula C = ɛ ɛ_0_ S/d. Consequently, a substantial surface area is inherently required for charge storage. The correlation of SSA vs. capacitance is in accordance with *Platek et al*.^[^ ^21^ ^]^ emphasizing that in both cases the materials prepared by the soft‐salt templating method, but with different salts, lead to similar results. However, non‐linear dependence has also been reported. As mentioned by *Kötz et al.* ^[^ ^25^ ^]^ the specific capacitance of selected commercial carbons does not show linearity with increasing the SSA over the entire range of values. Specifically, it exhibits a plateau for the values of SSA higher than 1200 m^2^ g^−1^. This behavior is related to the space constriction for the charge accommodation inside the pore walls (>1200 m^2^ g^−1^; the pore walls can no longer accommodate the same amount of the charge at a given electrode potential). It is worth mentioning that the authors used organic electrolytes, which present larger molecular size contrary to our studies focused on aqueous electrolytes. In addition, there is no information about the degree of disorder and surface functionalities of the selected carbons, which surely impacts the performance of EDLCs. The gravimetric capacitance in aqueous electrolyte was found as almost proportional to SSA in a series of carbons with progressively modified textural properties by heat treatment at different temperatures, followed by KOH activation.^[^ ^29^ ^]^ Moreover, the analysis of electrochemical performance in various electrolytes revealed that adequate pore size is a mandatory requirement to benefit from the high surface area, otherwise, the SSA cannot be fully accessible to the ions. *Lobato et al*. utilized a variety of carbons and found that standard textural characterization by gas adsorption may be very limited in evaluating their actual porosity involved in charge storage.^[^ ^23^ ^]^ Selecting the proper approach for gas sorption is crucial to finding the real SSA and pore size distribution. Nevertheless, SSA from the isotherm sorption will never reflect the electrochemically available surface area.

Interestingly, the effective specific surface area (ESSA) is proposed as a link between the textural properties of carbon and storage behavior.^[^ ^86^ ^]^ The term ESSA is known also as an accessible specific surface area. It should be emphasized that the SSA determined by the physisorption of the standard N_2_ molecule may not access all the pores (small micropores, called ultramicropores), therefore, CO_2_ sorption is used as a complementary technique to estimate the volume of ultramicropores (<0.7 nm). These narrow pores are extremely important for EDLCs operating in aqueous medium since desolvated and solvated ions are much smaller than organic/ionic liquid ions. In our series of carbons, a fully accessible specific surface area is involved in charge storage, thus a linear correlation between surface area and capacitance is reported. Therefore, tailoring carbon materials by the so‐called matching of the average diameter of micropores with solvated ion dimensions cannot be neglected. Additionally, small mesopores are advantageous for effective dynamic charge propagation and must be taken into account as well.

In Figure 8e,f the average diameter of micropores and mesopores is plotted vs. the gravimetric capacitance at 1 A g^−1^. A linear dependence is visible in both cases with R^2^ = 0.77 (L_0 micro_) and R^2^ = 0.85 (L_0 meso_). A slight deviation from the linear trend is observed for L_0 micro_ vs. gravimetric capacitance for LiCl/NaCl‐T carbon. The lowest volume of micropores among all carbons for LiCl/NaCl‐T may be caused by the highest ordering of the structure. The capacitance increases linearly with L_0 micro_, whereas diminishes in the case of higher mesopores. This illustrates that the gravimetric capacitance increases with the increase of micropore size up to 1 nm (the studied range), while for the mesopores, the smaller pores (≈4 nm) are more efficient than the larger pores (5–8 nm). Therefore, the correlation reported herein does not support the anomalous increase of capacitance for pores below 1 nm.^[^ ^26^ ^]^ The correlation of capacitance retention vs. L_0 micro_ (Figure S15b, Supporting Information) and L_0 meso_ (Figure S15c, Supporting Information) shows good linear dependence with R^2^ coefficients of 0.73 and 0.75, respectively. Another factor of importance is the total volume of pores, Figure S15d–f (Supporting Information) shows the correlation of gravimetric capacitance (1 A g^−1^, 1.6 V) and V_ultramicro_ (d<0.7 nm), V_micro_ (d<2.0 nm), and V_meso_ (d>2.0 nm), respectively. Despite the discrepancy in volume of the smallest pores measured by distinct adsorbates (N_2_ and CO_2_), the trend in gravimetric capacitance is the same, i.e., with the highest volume of ultramicropores (Figure S15d, Supporting Information) and micropores (Figure S15e, Supporting Information) the capacitance is increasing with R^2^ 0.92 and 0.96, respectively. On the other hand, the relationship of V_meso_ and gravimetric capacitance (Figure S15f, Supporting Information) is the opposite, a smaller volume of mesopores matches with higher capacitances, however, the R^2^ is not as high (0.52) as in the case of microporous volume. Additionally, capacitance retention at a high current regime (20 A g^−1^) vs. V_micro_ (Figure S16a, Supporting Information), and V_meso_ (Figure S16b, Supporting Information) were plotted, respectively and the relationship is similar to the correlations with gravimetric capacitance at 1 A g^−1^ (1.6 V). It clearly shows that mesopores, especially bigger ones, are useless for efficient charge storage.

## 3 Conclusions

This extensive research offers substantial insights into the importance of tailoring the structure and texture of carbons for enhanced performance of aqueous‐based EDLCs. For that purpose, several physicochemical and electrochemical techniques were employed. The optimized salt templating synthesis allowed the preparation of porous carbons with different characteristics. Utilization of alkali metal chloride salts in their eutectic ratio enabled strict control of their melting points, thus ensuring the proper formation of local carbon structure and porosity. The electrochemical investigation of EDLCs based on such a prepared series of carbons and 1M Li_2_SO_4_ revealed good performance, with the highest capacitance of 244 F g^−1^ at 0.2 A g^−1^ (CsCl/KCl‐T) and specific energy over 10 Wh kg^−1^ observed in the wide power range at 1.6 V. The longest floating time was reported for LiCl/KCl‐T (288 h), while the shortest was for CsCl‐T (30 h)‐based EDLCs. These results emphasized that the most disordered material, highly microporous, with O‐functionalities, is prone to faster degradation. Controversially, the material that is more graphitic, and less microporous, thus with a lower amount of surface functionalities has enhanced long‐term performance.

The investigation of Raman spectra of salt‐templated carbons has been instrumental in elucidating the local ordering of a series of prepared carbons. Interestingly, the linear dependence of the degree of disorder and gravimetric capacitance of 1M Li_2_SO_4_‐based EDLCs has been found. It was revealed that salt‐templated carbons displaying higher disorder, namely those with a higher I_D_/I_G_ ratio, exhibited an increase of gravimetric capacitances at a wide range of current densities. Such findings were supported additionally by the active surface area determination. ASA related to the area occupied by the carbon structural defects presented quite a good correlation with gravimetric capacitances.

Another factor of importance is related to the porosity of salt‐templated carbons, i.e., specific surface area and pore size distribution (volume and average diameter of micropores and mesopores). The salt‐templated carbons were highly microporous with a specific surface area ranging from 2640 m^2^ g^−1^ for CsCl‐T to 1453 m^2^ g^−1^ for LiCl/NaCl‐T. The presence of the mesopores for more ordered carbons (LiCl templated) was also noted since they influence the ion transport. Importantly, a substantial specific surface area is inherently required for the formation of an electrical double layer, thus storing energy in the EDLC. However, the matching of the solvated ion size with the pore size (which has to be bigger or equal to the ion dimension) is the real factor that contributes to the enhanced performance of the EDLC.

Due to the possible interactions of the aqueous medium, which was utilized as an electrolyte for EDLCs with the carbon, the type and quantity of surface functionalities were investigated in detail, to prove the reliability of the provided correlations. Temperature‐programmed desorption coupled with mass spectrometry (TPD‐MS) allows for monitoring of CO and CO_2_ evolution and their deconvolution was matched with temperatures of decomposition of specific oxygen functional groups. The quantity of phenol/ether and carboxyl was substantial, thus showing linear dependence with gravimetric capacitance.

In conclusion, several parameters impact the performance of carbon‐based EDLCs, i.e., degree of disorder, specific surface area, pore size distribution, matching of size/kind of ions used, and surface functional groups. None of these should be ignored. In particular, the role of such parameters must be considered during the selection of the electrode material to achieve the desired EDLC performance. For instance, more disordered carbon, with accessible microporosity matching the solvated ions of the selected electrolyte, but also the presence of some mesoporosity (fast kinetics) and balanced surface functionalities content lead to enhanced gravimetric capacitance but a shorter cycle life. Contrarily, the ordered carbon material with an elevated amount of graphitic domains presents a long‐cycling life. In brief, even if highly disordered carbons supply enhanced capacitance, yet, defects are responsible for lifespan shortage.

## Conflict of Interest

The authors declare no conflict of interest.

## Author Contributions

A.K. performed conceptualization, data curation, formal analysis, investigation, methodology, visualization, and software, and wrote the original manuscript. B.R. performed data curation, visualization, and software. C.M.G.: performed conceptualization, funding acquisition, project administration, supervision, and validation, wrote, reviewed, and edited the final manuscript. E.F. performed conceptualization, funding acquisition, project administration, supervision, and validation, and wrote, reviewed, and edited the final manuscript.

## Supporting information



Supporting Information

## Data Availability

The data that support the findings of this study are available in the supplementary material of this article.
