# Supplementary material for: Impact of Disorder, Porosity, and Surface Chemistry of Salt Templated Carbons on Capacitance
Source: Adv Sci (Weinh). 2025 May 30;12(30):e05032. doi: 10.1002/advs.202505032 (PMC12376531; doi:10.1002/advs.202505032)
Supplement: Supplementary file 1 — Supporting Information [file ADVS-12-e05032-s001.docx]

**Supplementary Information**

**Impact of Disorder, Porosity and Surface Chemistry
of Salt Templated Carbons on Capacitance**

Amelia Klimek^1,2,3^, Bénédicte Réty^2,3^, Camélia Matei Ghimbeu*^2,3,4^, Elzbieta Frackowiak*^1^

^1^Poznan University of Technology, Institute of Chemistry and Technical Electrochemistry, Berdychowo 4, Poznan 60-965, Poland

^2^Université de Haute-Alsace, Institut de Science des Matériaux de Mulhouse, F-68100 Mulhouse, France

^3^Université de Strasbourg, F-67081 Strasbourg, France

^4^Réseau sur le Stockage Electrochimique de l’Energie (RS2E), 80039 Amiens Cedex, France

*Corresponding authors

[camelia.ghimbeu@uha.fr](mailto:camelia.ghimbeu@uha.fr), [elzbieta.frackowiak@put.poznan.pl](mailto:elzbieta.frackowiak@put.poznan.pl)

**Methods**

**Materials**

Chemical reagents used for synthesis of salt templated carbons, i.e., phloroglucinol, glyoxylic acid monohydrate, Pluronic F127 [poly(ethylene oxide)*-block*-poly(propylene oxide)*-block-*poly(ethylene oxide, PEO_106_PPO_70_PEO_106_, M_w_=12,600 Da)], sodium hydroxide, sodium chloride, lithium chloride, potassium chloride, caesium chloride, as well as lithium sulphate, were provided from Sigma Aldrich (99% purity). For electrode preparation, polytetrafluoroethylene (60 wt. % dispersion in water) was supplied by Sigma Aldrich, carbon black (C65) was provided by Imerys, and glass fibre GF/A, which served as separator, was given by Whatman.

**Salt templated carbons preparation**

A series of carbon materials with different textures and structures were prepared by applying the soft-salt templated method. Such a method enables perfect development of micropores due to the small size of selected cations and anions present in inorganic salt (salt template) and formation of mesoporosity due to decomposition of soft template. The soft-salt templated method has already been described in-detail^[1–3]^. The synthesis of salt templated carbon included the preparation of environmentally friendly phenolic resin. Carbon source (phloroglucinol, 1.66 g) and cross-linker (glyoxylic acid, 1.56 g) were dissolved in 20 cm^3^ of ethanol, then mixed with 40 cm^3^ of water. To the same solution, a soft template (Pluronic F127, 3.2 g) was added. It facilitated the development of micelles, which were subsequently combined with the phenolic resin (the carbon source) *via* hydrogen and/or covalent bindings to create a macromolecular structure. In the meantime, 20 cm^3^ of selected salt template solution was added to the reaction volume. Then, 16 cm^3^ of 1.25M sodium hydroxide was used to control the pH (~5). The objective of the work was to employ a double alkali metal chloride mixture in eutectic ratio, i.e., caesium chloride/sodium chloride (CsCl/NaCl), caesium chloride/potassium chloride (CsCl/KCl), caesium chloride/lithium chloride (CsCl/LiCl), sodium chloride/potassium chloride (NaCl/KCl), lithium chloride/sodium chloride (LiCl/NaCl), lithium chloride/potassium chloride (LiCl/KCl). The chloride salts used in their eutectic ratio ensure that salts will melt in total while applying elevated temperature during carbonisation step. Additionally, single soft-salt templated carbon using caesium chloride (CsCl) was utilised as a reference material for our findings^[1]^. Detailed information about used eutectic mixtures, melting points, and ratios is summarised in **Tab. S1**.

**Tab. S1** Parameters used during synthesis of salt templated carbons (eutectic salt mixture, chloride 1, chloride 2, melting point, ratio chloride 1: chloride 2 (%)).

| **Material** | **Chloride 1** | **Chloride 2** | **Tm (°C)** | **Ratio (%)** | **Reference** |
| --- | --- | --- | --- | --- | --- |
| CsCl-T | CsCl | - | 645 | 100 | ^[1]^ |
| CsCl/KCl-T | CsCl | KCl | 610 | 35/65 | ^[4]^ |
| CsCl/NaCl-T | CsCl | NaCl | 466 | 65/35 | ^[5]^ |
| CsCl/LiCl-T | CsCl | LiCl | 327 | 42/58 | ^[6]^ |
| NaCl/KCl-T | NaCl | KCl | 685 | 50/50 | ^[7]^ |
| LiCl/KCl-T | LiCl | KCl | 355 | 58.5/41.5 | ^[7]^ |
| LiCl/NaCl-T | LiCl | NaCl | 557 | 77.5/22.5 | ^[7]^ |

The molar ratio of 2:1 was kept between the salt mixture and the phenolic resin. The resultant solution was placed in multiple Petri plates and allowed to evaporate overnight at ambient temperature. Then, the polymer/salt assembly was heated to 120°C for 6 h to cross-link the phenolic resin, and it was annealed in argon flow for 1 h at 900°C (heating rate 2°C min^-1^) to obtain the carbon/salt material. To remove the chloride salts, the as-obtained material was washed using approximately 1 L of hot (80°C) distilled water, while the pH was controlled (~7). The final material was dried at 80°C overnight and manually ground in the mortar. For clarification, in the main body of the manuscript, the prepared materials are called salt templated carbons since the parameter that was changed is the type of salt template, while the soft template remained constant. Moreover, prepared carbons were labelled, according to the **Tab. S1**, to distinguish the inorganic salts (i.e., CsCl/NaCl-T), where T represents the templated carbon.

**Physicochemical characterization of the materials**

The X-ray diffraction was used to characterize the salt templated carbon structure using a D8 Advance A25 diffractometer from Burker in the θ−θ Bragg-Brentano reflection geometry equipped with a Lynx Eye XE-T high resolution energy dispersive 1D detector (CuKα_1,2_) that enabled ultra-fast X-ray diffraction measurements (angular area 3-90º2θ, step size 0.017º2θ, time per step 1.8s, variable divergence slit mode). The local material structure was analysed with transmission electron microscopy (JEOL ARM-200F that was operating at 200 kV). Complementary information of carbon local structure and defects were obtained by Raman spectroscopy performed with a LabRAM 300 instrument from Horiba Jobin-Yvon, which was equipped with a solid-state laser (Compass 315M from Coherent, wavelength 532 nm). The power of the laser was adjusted to 1.2 mW. Each Raman spectrum was obtained after collecting 11 scans at different points on the sample, each acquisition being performed in 10 s. For peak evaluation, the average Raman spectrum (after 11 scans over each sample) was normalized by the maximum intensity, baseline subtracted and deconvoluted into two peaks using the Lorentzian fitting function through the peak analyser tool available in Origin v9.65 (OriginLab). Two different deconvolution methods were used for data fitting: with two-peaks and with four-peaks, as described below. For two-peaks fitting, the initial peak positions were used: D ~ 1350 cm^-1^, G ~1580 cm^-1^, then freely adjusted to obtain the optimum fit. The I_D_/I_G_ ratio was calculated using both the fitted D and G peak areas (I_D_/I_G_ area) and the intensities of the D and G peaks (I_D_/I_G_ intensity). For four-peaks evaluation, the initial peak positions were used: D_1_ ~ 1350 cm^-1^, D_2_ ~ 1180 cm^-1^, D_3_ ~ 1500 cm^-1^, G ~1580 cm^-1^, then freely adjusted to attain optimal fit (the positions of each peak cannot be set as constant, each spectrum is different, due to the differences of salt templated carbons texture/structure, therefore negligible shifts could be observed)^[8,9]^. The parameters obtained by the two- and four-peaks fitting, including fitted peak areas, full width at half maximum and peak intensities were used for in-detail analysis/correlations. The I_D1_/I_G_ ratio was calculated using the fitted D_1_ and G peak areas (I_D1_/I_G_ area) and intensities of the D_1_ and G peaks (I_D1_/I_G_ intensity). Additionally, I_D2_/I_G_ and I_D3_/I_G_ ratios were calculated in the same manner as I_D1_/I_G_. Each spectrum was deconvoluted at least three times and the standard deviation among the fits was calculated.

The nitrogen and carbon dioxide sorption measurements were performed with a Micromeritics ASAP 2020 device at 77K and 273K, respectively. The salt templated carbons were outgassed in vacuum at 300°C for 12 h before sorption analysis. The specific surface area was calculated by the Brunauer-Emmett-Teller (BET) model from the linear dependence at relative pressures ranging from 0.01 to 0.05 (N_2_) and 0.01 to 0.03 (CO_2_). The micropore volume was obtained using the Dubinin-Radushkevich equation in the relative pressure interval (p/p_0_) from 10^-4^ to 10^-2^. The pore size distribution was determined from the adsorption branch of nitrogen isotherms using a 2D-NLDFT heterogeneous surface model for carbon materials implemented in SAIEUS (Micromeritics). The determination of micropores/mesopores average diameter was performed in accordance with the reference^[1]^.

Elemenal analysis (Thermo Scientific^TM^ FlashSmart^TM^ Elemental Analyser, USA) was performed to investigate the % content of carbon, hydrogen, nitrogen, and oxygen in salt templated carbons. The quantity of oxygen was determined *via* direct elemental analysis. The presented results are the average values of three separate analyses.

The temperature programmed desorption combined with mass spectrometry was used to evaluate the active surface area and surface chemistry of carbons. The salt templated carbon was heated (5°C min^-1^) up to 950°C in a vacuum (10^-7^ Torr). Then, oxygen-containing functional groups break down into CO and CO_2_, as well as H_2_ and H_2_O, which are continually monitored by a mass spectrometer. By performing calibration of each individual pure gas prior to the samples’ analyses it is then possible to calculate their numbers of moles. Each CO_2_ and CO curve was deconvoluted to determine exact type and amounts of oxygen functionalities (carbonyl/quinone, phenol/ether, lactone, anhydride, and carboxylic)^[10]^. Indeed, these surface groups decompose as CO and/or CO_2_ at various temperatures^[11,12]^. The deconvolution of the TPD-MS CO_2_ profile gives the amounts of carboxylic acids, anhydrides, and lactones, while the deconvolution of the CO profile is used to quantify the phenols-ethers, and carbonyl-quinones groups. The deconvolutions were performed according to the *Ishii et al.*^[10]^ and *Rocha et al.*^[12]^ procedures. All the deconvolution parameters are given in one of our previous work^[13]^. The deconvolution of the CO_2_ and CO TPD-MS desorption profiles allows to quantify the different oxygenated surface groups of the carbon materials. It starts with the deconvolution of the CO_2_ profiles which is the result of the decomposition of carboxylic acids (CO_2_ < 450°C), anhydrides (CO_2_+CO: 350-600°C) and lactones (CO_2_ > 600°C). Carboxylic acids are deconvoluted in two peaks which are due to weak and strong acids. The CO desorption rate profile is due to the contribution of anhydrides (CO_2_+CO: 350-600°C), phenols-ethers (CO: 500-750°C) and carbonyls-quinones (CO > 700°C). Anhydrides decompose as one mole of CO_2_ and one mole of CO. Because the quantity of anhydride has already been calculated from the CO_2_ profile, a similar peak (same peak position, full width at half maximum and area under the curve) is defined for the CO profile and allows to consider its contribution to the release of CO. A Gaussian-type fit and peak analyser tool of Origin Pro were used.

After the removal of surface functionalities from carbon during the first TPD-MS experiment, the active surface area was determined by exposing the material to di-oxygen at 150°C for 10 h, according to the procedure described elsewhere^[13]^. To quantify the newly generated surface oxygen functionalities resulting from the interaction of carbon defects, i.e., active sites with oxygen, a second TPD-MS, carried out up to 950°C at 10°C min^-1^. By the integration of obtained gas desorption profiles and the calibration of these pure gases, the number of CO and CO_2_ moles, which come from the thermal decomposition of these oxygenated functionalities, is measured. Then, using the Avogadro’s constant, the number of oxygen atoms involved in the interactions with active sites can be determined. *Laine* *et al.*^[14]^ showed that the area of an edge carbon site, i.e., active site, on which an oxygen atom is chemisorbed is equal to 0.083 nm^2^. Therefore, the surface area covered by the oxygen chemisorbed on all active sites, also called the active surface area, is calculated according to the following equation^[15]^:

$ASA=\left( n_{co}+2n_{co_{2}} \right) A*N_{A}{[m}^{2} g^{-1}]$

where: n – number of moles (mol g^-1^), A – average area of an edge carbon atom (0.083 nm^2^ = 8.3 * 10^-20^ m^2^), N_A_ – Avogadro constant (6.022 * 10^-23^ mol^-1^).

The wettability analysis was conducted using the DSA100, Krüss equipped with goniometer. As the liquid, 1M Li_2_SO_4_ was used with a volume of 1.8 µL. Each measurement of contact angle value was performed for 30 seconds and repeated six times. The final contact angle value is the average value of all the measurements.

**Electrochemical characterization of the materials**

Self-standing electrodes were prepared by physically grinding and mixing 90 wt.% of salt templated carbon, 5 wt.% of polytetrafluoroethylene binder (PTFE; 60 wt.% dispersion in water), and 5 wt.% of carbon black (C65) together with some isopropanol in a mortar during approximately 30 minutes. The obtained dough-like material was rolled into an electrode sheet, which was calendared between two polyethylene films, until it reached a thickness of 210 – 250 µm. Self-standing electrodes were cut from the sheet using a hollow punch to reach 10 mm in diameter (mass loading 7 – 8 mg) and dried at 90°C overnight.

Two carbon electrodes separated by a glass fibre (12 mm diameter, 260 µm thickness) were soaked with 1M Li_2_SO_4_ electrolyte (pH = 8, conductivity = 72 mS cm^-1^) and placed in a symmetric PTFE-based Swagelok® system with stainless steel 316L current collectors. For three electrode measurements Hg|Hg_2_SO_4_ was used as the reference electrode. A VMP3 multi-channel potentiostat/galvanostat (BioLogic, France) was used to investigate the electrochemical performance of EDLCs at room temperature. Techniques such as cyclic voltammetry at scan rates 1 – 200 mV s^-1^, galvanostatic charge/discharge at current densities 0.2 – 20 A g^-1^, and electrochemical impedance spectroscopy at frequency range 100 kHz – 1 mHz were employed. Presented values of the gravimetric capacitances were calculated from cyclic voltammetry using the formula^[16]^:

$$C_{CV}=\frac{2 I}{v m_{a}} [F g^{-1}]$$

where: I – current, v – scan rate, m_a_ – active mass of the electrode and from galvanostatic charge/discharge results utilizing equations from ref^[16]^ as follows:

$E_{int/GD}=I\int_{t(U_{max)}}^{t(U_{min})} U\left( t \right)dt [W s]$

$$C_{int/GD}=\frac{2 E_{int/GD}}{U_{max}^{2}} [F]$$

$$C_{el/int,GD =}\frac{C_{int/GD}}{0.5m_{el}} [F g^{-1}]$$

where: E_int/GD_ is a discharge energy, C_int/GD_ is the capacitance calculated by integrating the area under the galvanostatic discharge curve, C_el/int,GD_ is the gravimetric capacitance calculated by integrating the area under the galvanostatic discharge curve expressed per one electrode, m_el_ is the total mass of both electrodes. The presented specific energy (E_s,int/GD_) and specific power (P_s,int/GD_) were calculated based on the following formulas^[16]^:

$$E_{s,int/GD}=\frac{E_{int/GD}}{m_{el} 3.6} [Wh {kg}^{-1}]$$

$$P_{s,int/GD}=\frac{E_{s,int/GD}}{t_{dis}} [W {kg}^{-1}]$$

where: t_dis_ represents a discharge time. The electrochemical performance data shown in the manuscript is based on three experimental runs. It is important to note that the gravimetric capacitance of a full device will be four times smaller than the one calculated per mass of one electrode^[17]^. Volumetric capacitance was calculated according to the equation:

$$C_{V}=C_{el/int GD}\frac{m}{V} [F {cm}^{-3}]$$

where: C_el/int,GD_ is the capacitance calculated by integrating the area under the galvanostatic discharge curve expressed per one electrode, m is the average mass of one electrode, V is volume of one electrode. Volume of one electrode was calculated using the formula:

$$V=\pi r^{2} h [cm^{3}]$$

where: r is the radius of one electrode, h is the thickness of one electrode^[18]^. The floating test was used to determine the EDLCs long-term stability. Three galvanostatic charges/discharges (1 A g^-1^) at the maximum, stable operating voltage (U_max_= 1.6 V) were followed by a voltage hold for 2 h at U_max_^[19]^. After each voltage hold the CV and EIS spectra were recorded. The procedure was repeated until one of the end-of-life criteria was reached, i.e., a 20% decrease in initial gravimetric capacitance or a 100% increase in initial resistance. Additionally, the self-discharge technique was used, consisting of GCD at 1 A g^-1^, U_max_ followed by 2 h hold and recording the open circuit voltage for 12 h.


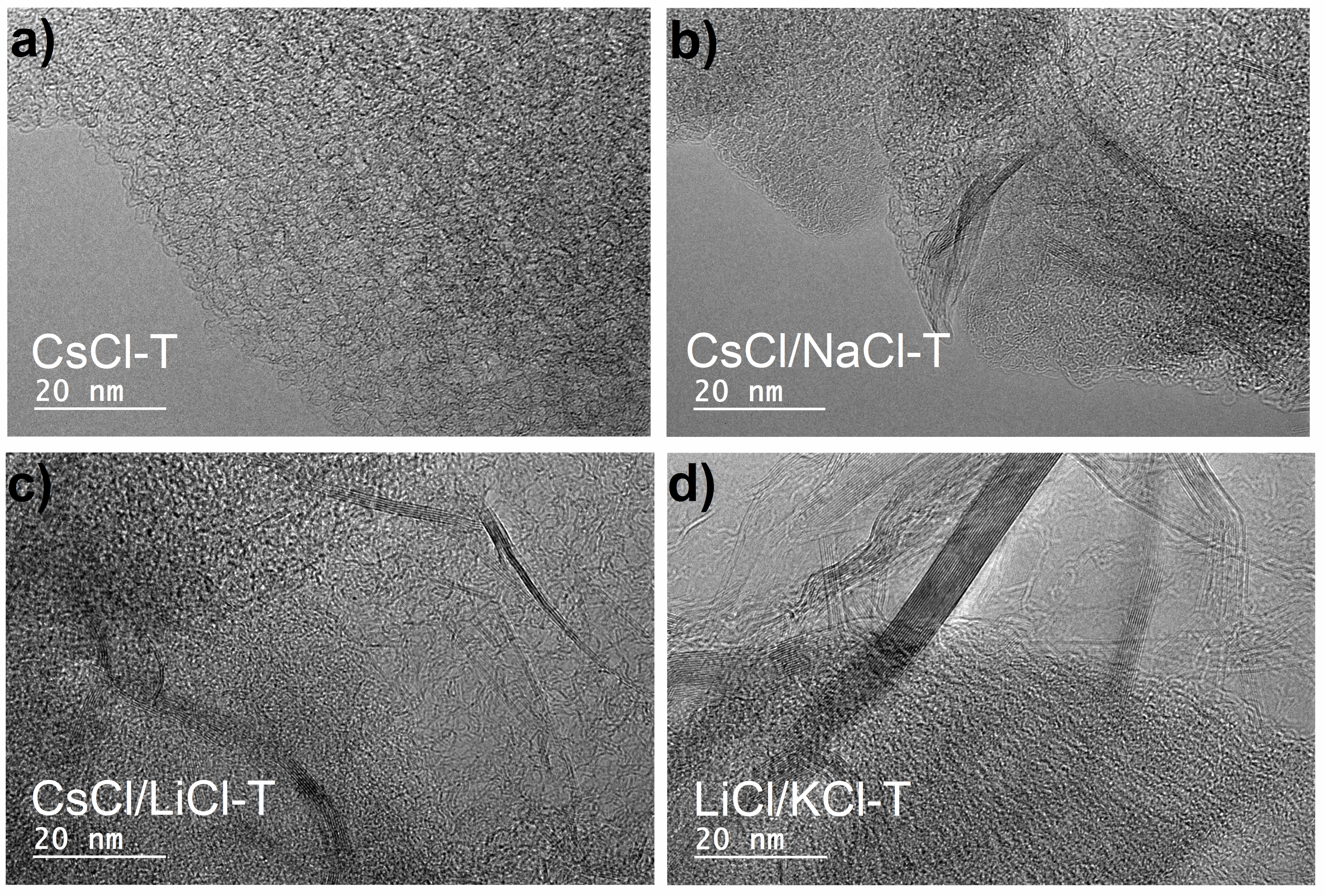


**Fig. S1** TEM images of selected salt templated carbons: **a)** CsCl-T, **b)** CsCl/NaCl-T, **c)** CsCl/LiCl-T,
**d)** LiCl/KCl-T.

**Tab. S2** Selected thermodynamic and physical characteristics of ions used in the synthesis of salt templated carbons and as the electrolyte for EDLC testing.

| Ion | Crystal ionic diameter  (nm) | Hydrated ion diameter  (nm) | Hydration enthalpy  (kJ mol^-1^) | Binding energy  (kcal mol^-1^) |
| --- | --- | --- | --- | --- |
| Li^+^ | 0.152^20^ | 0.680^21^ | 520^2^ | 38^2^ |
| Na^+^ | 0.232^20^ | 0.598^21^ | 406^2^ | 28^2^ |
| K^+^ | 0.304^20^ | 0.662^22^ | 322^2^ | 19^2^ |
| Cs^+^ | 0.362^20^ | 0.658^23^ | 276^2^ | 16^2^ |
| Cl^-^ | 0.338^22^ | 0.664^23^ | 378^24^ | - |
| SO_4_^2-^ | 0.430^25^ | 0.600^25^ | 1330^26^ | - |


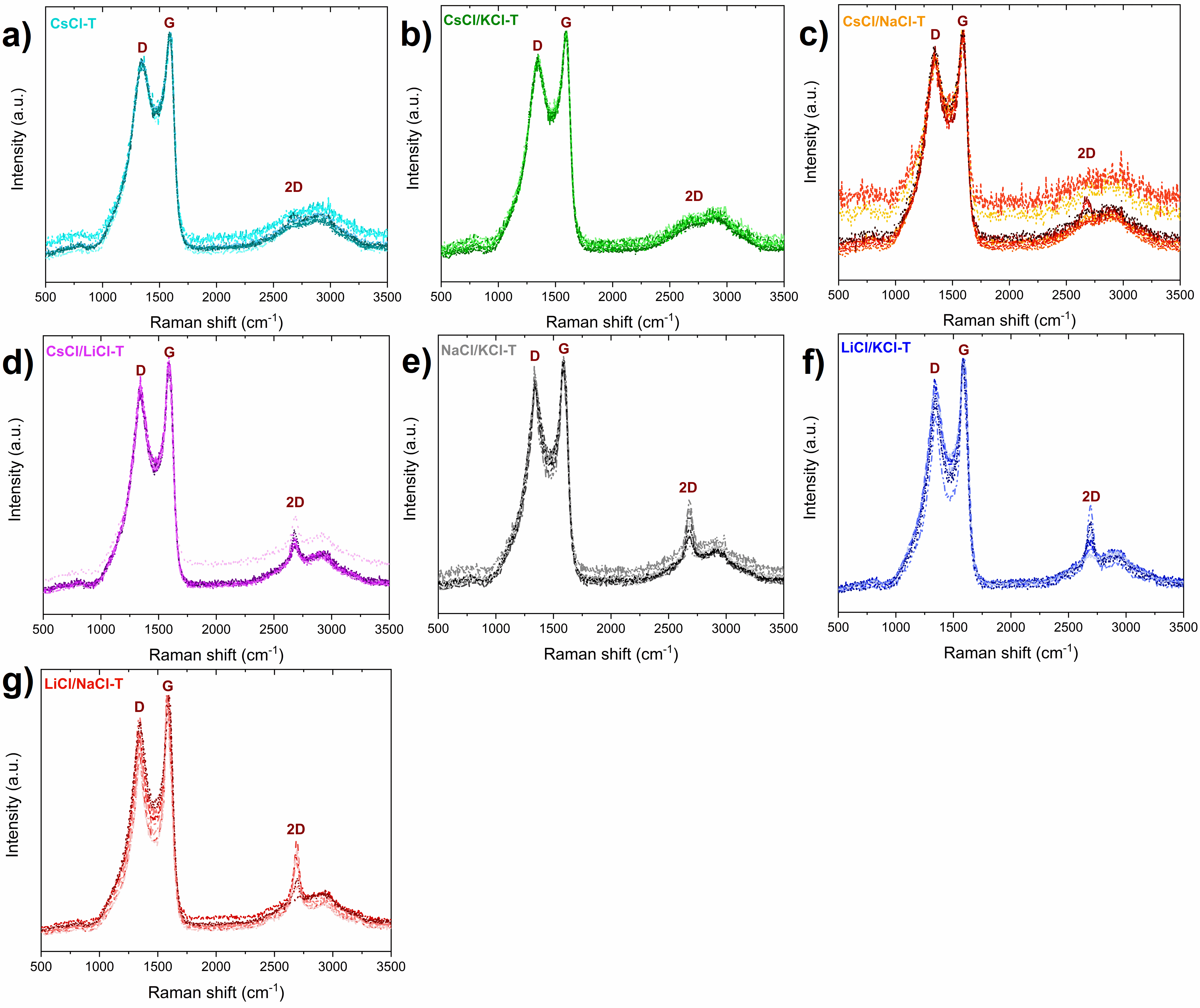


**Fig. S2** Normalized Raman spectra collected at 11 different places over the salt templated carbons:
**a)** CsCl-T, **b)** CsCl/NaCl-T, **c)** CsCl/KCl-T, **d)** CsCl/LiCl-T, **e)** NaCl/KCl-T, **f)** LiCl/NaCl-T, **g)** LiCl/KCl-T.


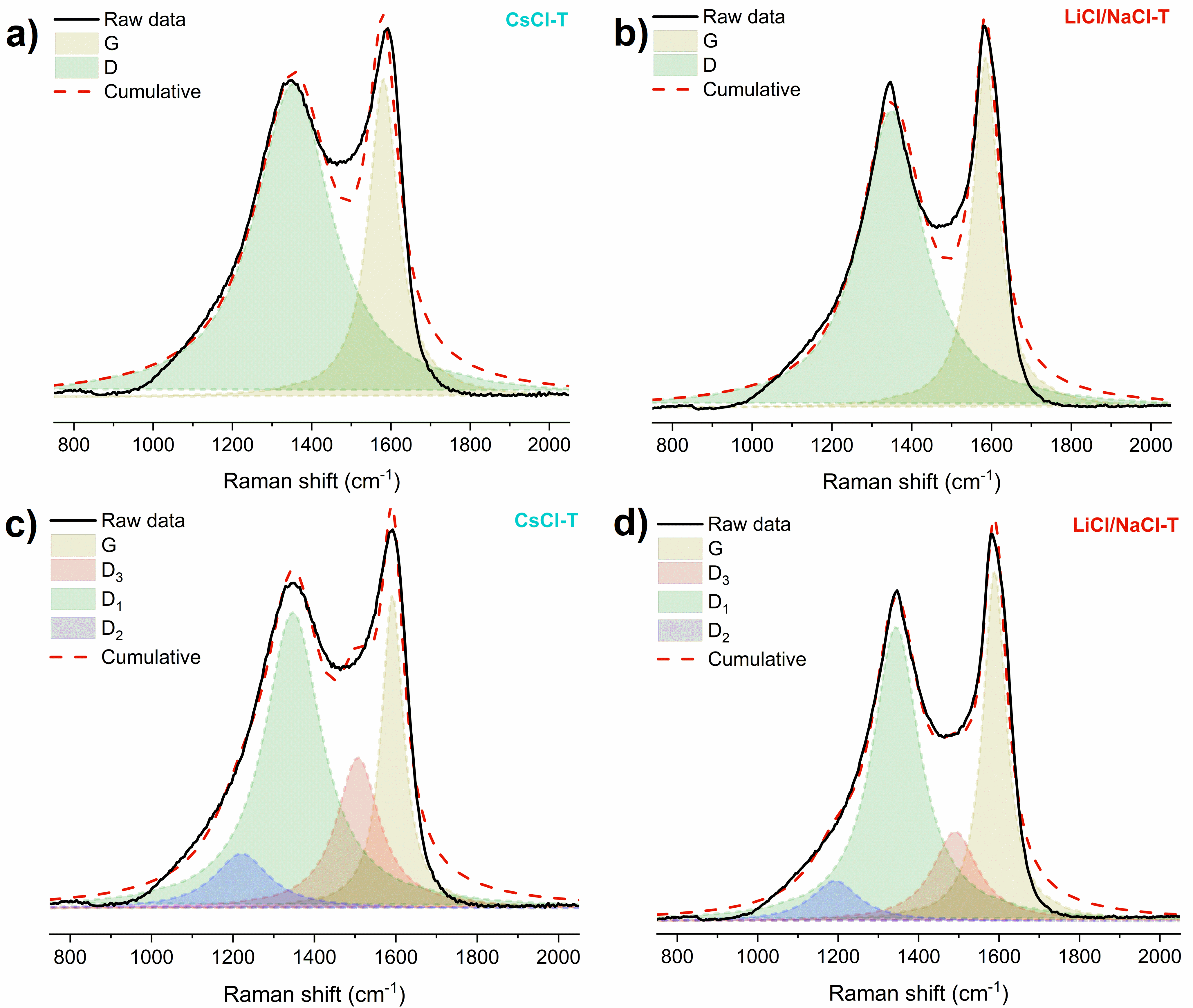


**Fig. S3** A comparison of Raman spectra deconvoluted into: (top) two peaks using Lorentzian fitting function for the **a)** most disordered CsCl-T and **b)** most ordered LiCl/NaCl-T salt templated carbons and (bottom) four peaks using Lorentzian fitting function for the **c)** most disordered CsCl-T and **d)** most ordered LiCl/NaCl-T salt templated carbons.

**Tab. S3** Summary of the spectral parameters of salt templated carbons after 2 peak fitting utilizing Lorentzian fitting function i.e., peaks area (D_,_ G), full width at half maximum (FWHM D), peak areas ratios related to the area of G band (I_D_/I_G_ area), peak intensities ratios related to the intensity of G band (I_D_/I_G_ intensity) and standard deviation among the fits.

| Material | D  area | G  area | FWHM  D | I_D_/I_G_ area | I_D_/I_G_ intensity |
| --- | --- | --- | --- | --- | --- |
| CsCl-T | 254.1  ±0.005 | 105.3  ±0.009 | 234.6  ±0.010 | 2.41  ±0.000 | 0.99  ±0.000 |
| CsCl/KCl-T | 249.6  ±0.098 | 105.6  ±0.123 | 229.4  ±0.138 | 2.36  ±0.004 | 0.98  ±0.002 |
| CsCl/NaCl-T | 242.0  ±1.967 | 107.6  ±1.592 | 221.3  ±2.678 | 2.26  ±0.051 | 0.97  ±0.007 |
| CsCl/LiCl-T | 225.3  ±0.068 | 107.1  ±0.101 | 210.1  ±0.101 | 2.20  ±0.003 | 0.92  ±0.000 |
| NaCl/KCl-T | 230.4  ±0.004 | 102.7  ±0.004 | 211.7  ±0.004 | 2.17  ±0.000 | 0.93  ±0.000 |
| LiCl/KCl-T | 230.4  ±0.003 | 106.0  ±0.003 | 211.2  ±0.003 | 2.16  ±0.000 | 0.89  ±0.000 |
| LiCl/NaCl-T | 224.1  ±2.924 | 103.3  ±0.349 | 197.3  ±2.646 | 2.01  ±0.201 | 0.85  ±0.002 |

**Tab. S4** Summary of the spectral parameters of salt templated carbons after 4 peak fitting utilizing Lorentzian fitting function i.e., peaks area (D_1_, D_2_, D_3_, G), full width at half maximum (FWHM D_1_), peak areas ratios related to the area of G band (I_D1_/I_G_ area), peak intensities ratios related to the intensity of G band (I_D1_/I_G_ intensity), full width at half maximum related to the area of G band (FWHM D_1_/G) and standard deviation among the fits.

| Material | D_1_  area | D_2_  area | D_3_  area | G  area | I_D1_/I_G_ area | I_D1_/I_G_ intensity | FWHM D_1_/G | FWHM  D_1_ |
| --- | --- | --- | --- | --- | --- | --- | --- | --- |
| CsCl-T | 176.1  ±8.273 | 31.5  ±9.263 | 68.1  ±0.651 | 74.7  ±1.202 | 2.36  ±0.075 | 0.95  ±0.010 | 2.59  ±0.013 | 171.9  ±2.687 |
| CsCl/KCl-T | 172.6  ±2.287 | 31.7  ±1.242 | 64.9  ±3.132 | 76.2  ±0.306 | 2.27  ±0.028 | 0.95  ±0.008 | 2.51  ±0.029 | 168.4  ±1.587 |
| CsCl/NaCl-T | 178.6  ±6.800 | 24.0  ±6.564 | 55.7  ±1.531 | 82.4  ±1.501 | 2.17  ±0.045 | 0.95  ±0.026 | 2.39  ±0.012 | 168.6  ±2.762 |
| CsCl/LiCl-T | 166.7  ±0.551 | 22.9  ±0.231 | 48.4  ±0.404 | 83.5  ±0.643 | 2.00  ±0.012 | 0.91  ±0.007 | 2.28  ±0.002 | 156.2  ±0.289 |
| NaCl/KCl-T | 163.0  ±0.700 | 26.8  ±0.100 | 53.6  ±0.321 | 86.5  ±0.265 | 1.88  ±0.009 | 0.90  ±0.000 | 2.14  ±0.004 | 151.0  ±0.152 |
| LiCl/KCl-T | 156.2  ±1.518 | 23.7  ±2.370 | 51.7  ±2.442 | 84.3  ±1.332 | 1.85  ±0.045 | 0.86  ±0.011 | 2.22  ±0.033 | 150.2  ±0.808 |
| LiCl/NaCl-T | 149.2  ±1.504 | 20.7  ±2.196 | 43.4  ±2.060 | 89.8  ±1.050 | 1.66  ±0.035 | 0.84  ± 0.006 | 2.03  ±0.024 | 143.5  ±2.706 |

**Tab. S5** Summary of the spectral parameters of salt templated carbons after 4 peak fitting utilizing Lorentzian fitting function i.e., full width at half maximum (FWHM D_2_, FWHM D_3_), peak areas ratios related to the area of G band (I_D2_/I_G_ area, I_D3_/I_G_ area), peak intensities ratios related to the intensity of G band (I_D2_/I_G_ intensity, I_D3_/I_G_ intensity), and standard deviation among the fits.

| Material | I_D2_/I_G_ area | I_D2_/I_G_ intensity | FWHM  D_2_ | I_D3_/I_G_ area | I_D3_/I_G_ intensity | FWHM  D_3_ |
| --- | --- | --- | --- | --- | --- | --- |
| CsCl-T | 0.42  ±0.200 | 0.18  ±0.057 | 165.1  ±0.424 | 0.91  ±0.090 | 0.47  ±0.027 | 128.5  ±3.111 |
| CsCl/KCl-T | 0.42  ±0.018 | 0.19  ±0.008 | 148.2  ±5.710 | 0.85  ±0.042 | 0.49  ±0.014 | 121.2  ±9.248 |
| CsCl/NaCl-T | 0.29  ±0.088 | 0.14  ±0.026 | 150.0  ±9.686 | 0.68  ±0.032 | 0.42  ±0.008 | 116.9  ±0.493 |
| CsCl/LiCl-T | 0.27  ±0.004 | 0.13  ±0.006 | 147.7  ±0.100 | 0.58  ±0.009 | 0.35  ±0.003 | 116.2  ±0.839 |
| NaCl/KCl-T | 0.31  ±0.001 | 0.14  ±0.007 | 158.3  ±0.115 | 0.62  ±0.006 | 0.38  ±0.000 | 120.4  ±0.700 |
| LiCl/KCl-T | 0.28  ±0.023 | 0.12  ±0.018 | 144.2  ±3.714 | 0.61  ±0.038 | 0.33  ±0.002 | 130.0  ±7.731 |
| LiCl/NaCl-T | 0.23  ±0.022 | 0.12  ±0.006 | 144.6  ±6.293 | 0.48  ±0.028 | 0.25  ±0.012 | 137.0  ±9.786 |


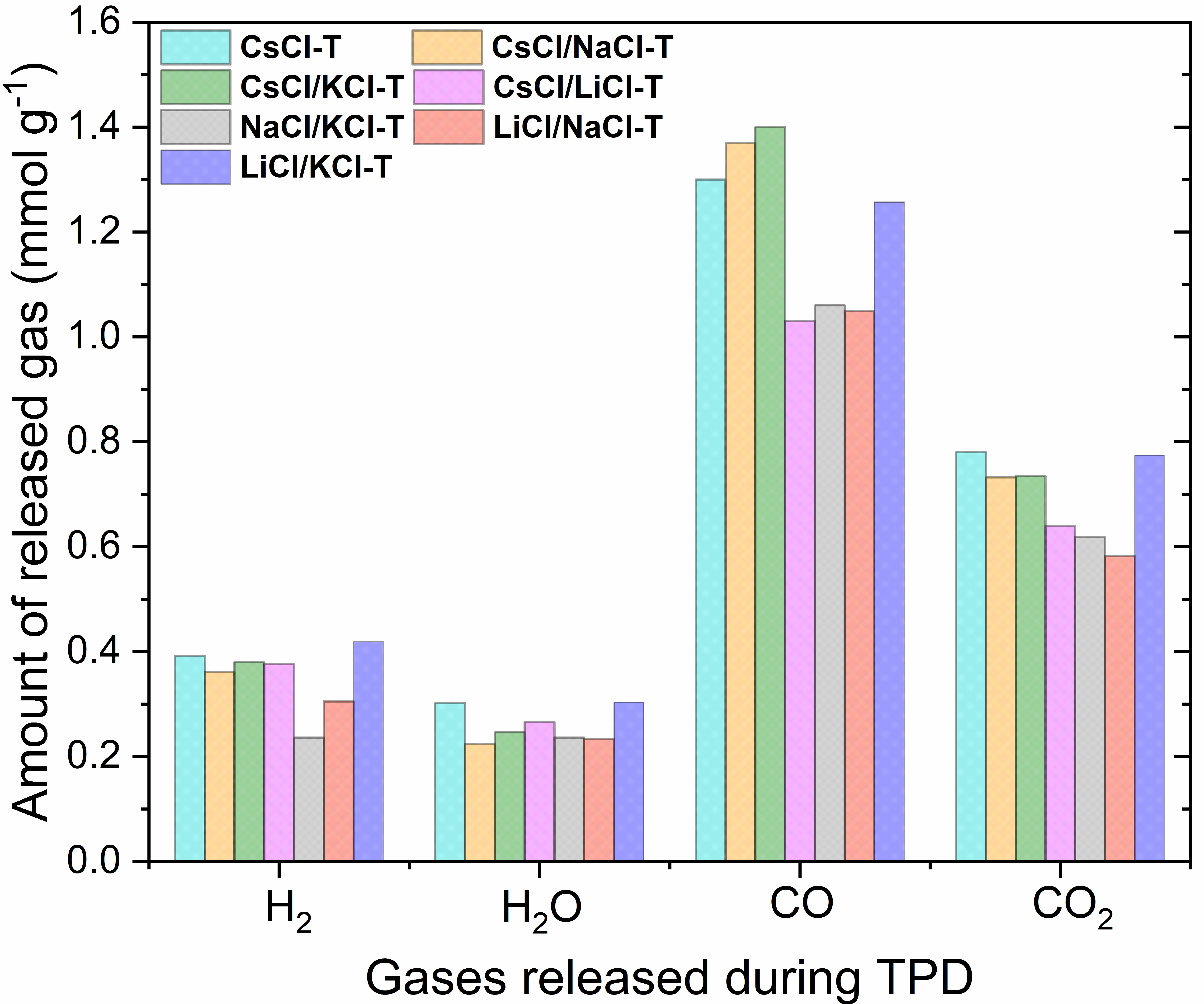


**Fig. S4** Amounts of H_2_, H_2_O, CO, and CO_2_ released, obtained by integration of the TPD-MS peaks of salt templated carbons.


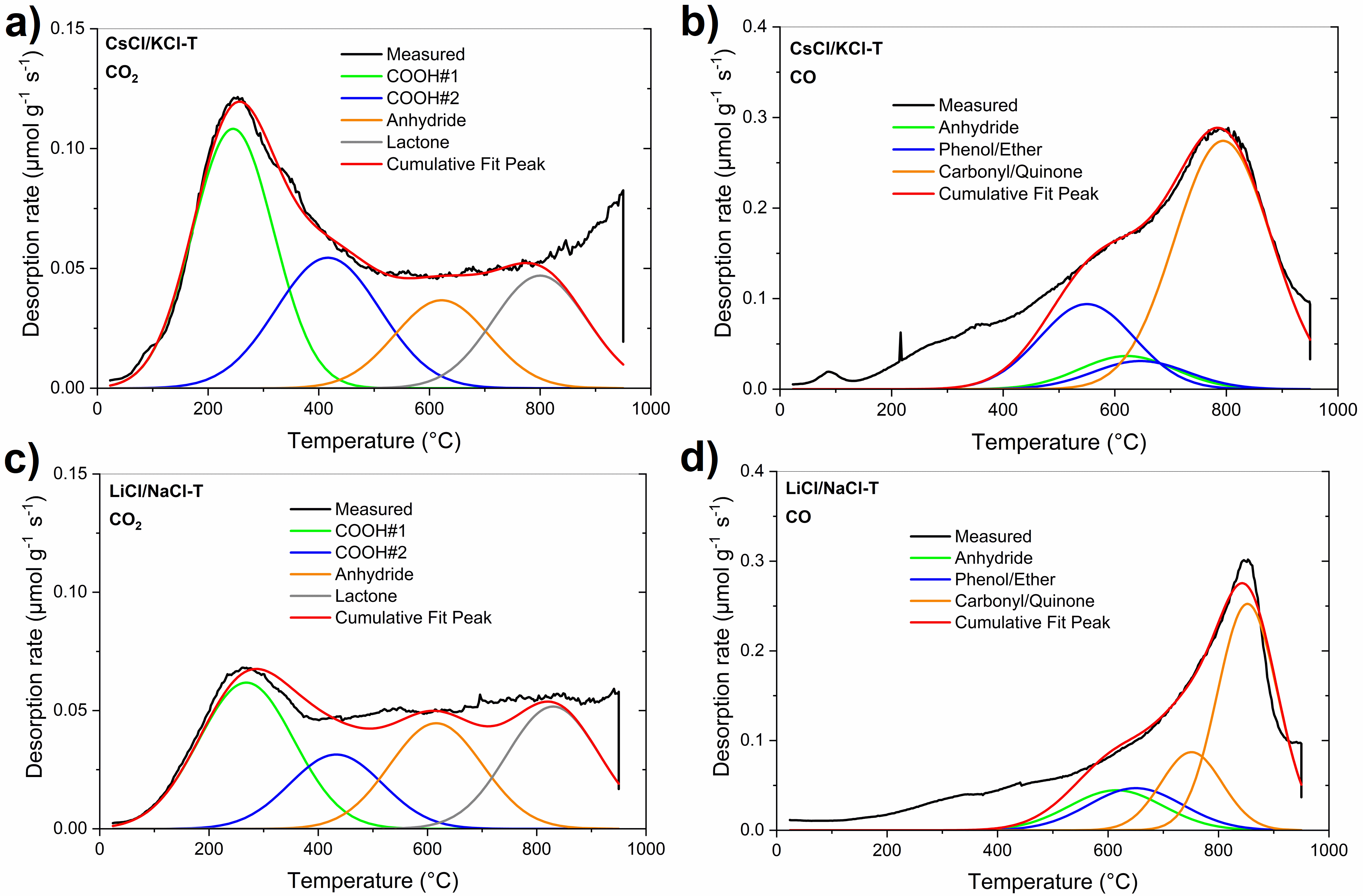


**Fig. S5** Deconvolution of TPD-MS gas desorption profiles (CO, CO_2_) for selected salt templated carbons: **a-b)** CsCl/KCl-T, **c-d)** LiCl/NaCl-T.

**Tab. S6** Textural properties determined by carbon dioxide sorption at 273K (SSA, V_ultramicropores_) of
salt templated carbons.

| Salt templated carbon | SSA  (m^2^ g^-1^) | V ultramicropores  (cm^3^ g^-1^) |
| --- | --- | --- |
| CsCl-T | 1361 | 0.79 |
| CsCl/KCl-T | 1409 | 0.79 |
| CsCl/NaCl-T | 1303 | 0.77 |
| CsCl/LiCl-T | 1186 | 0.70 |
| NaCl/KCl-T | 1152 | 0.68 |
| LiCl/KCl-T | 942 | 0.55 |
| LiCl/NaCl-T | 914 | 0.52 |


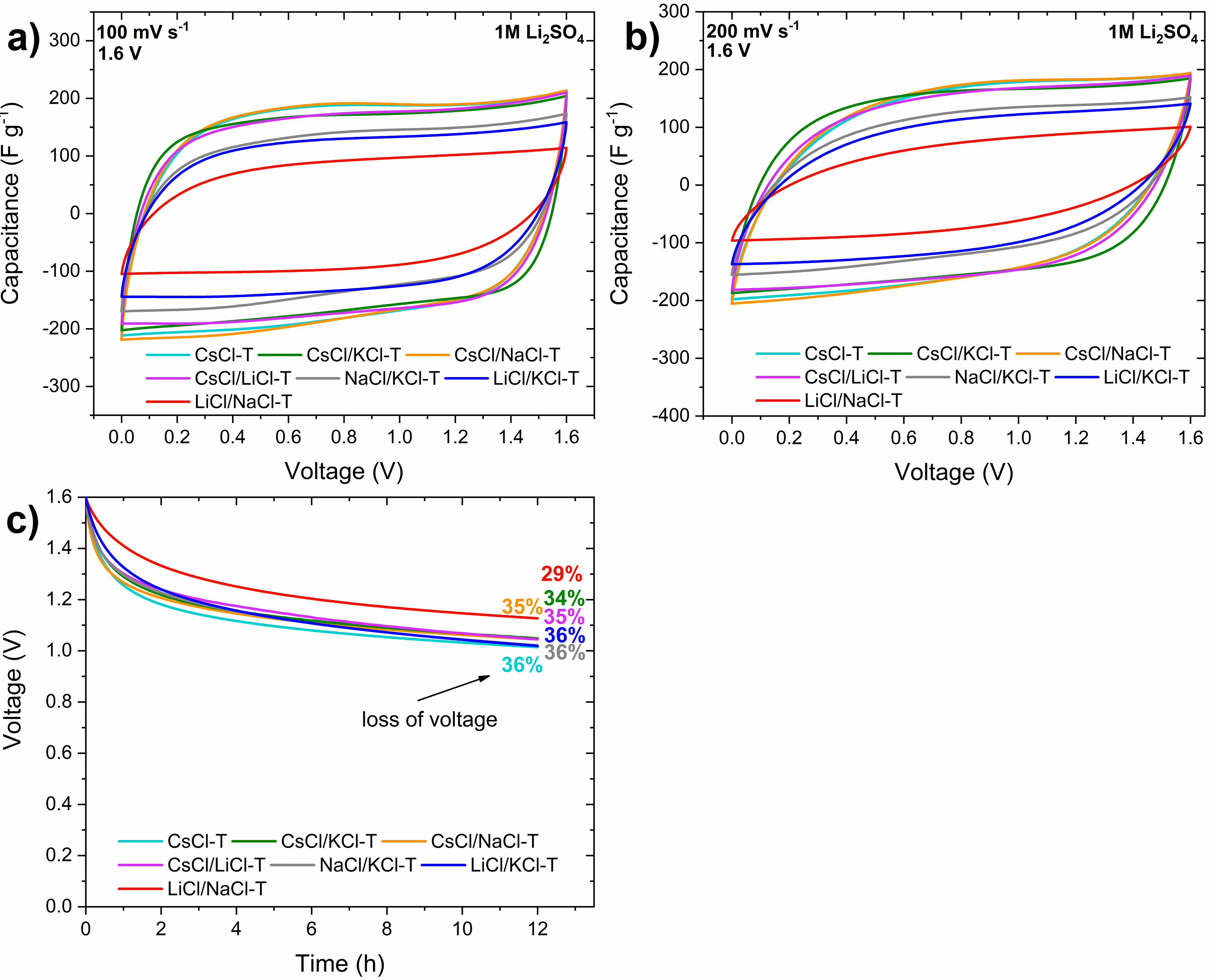


**Fig. S6** Electrochemical performance salt templated carbons-based EDLCs operating in 1M Li_2_SO_4_ at 1.6 V: **a)** cyclic voltammetry at 100 mV s^-1^, **b)** cyclic voltammetry at 200 mV s^-1^.

**Tab. S7** Summary of EDLCs performance based on different carbons operating in aqueous medium.

| Carbon | Electrolyte | Conditions | Gravimetric  capacitance | Reference |
| --- | --- | --- | --- | --- |
| CsCl-T | 1M Li_2_SO_4_ | 1.6 V  1 A g^-1^ | 197 F g^-1^ | This work |
| CsCl/KCl-T |  |  | 190 F g^-1^ |  |
| CsCl/NaCl-T |  |  | 181 F g^-1^ |  |
| CsCl/LiCl-T |  |  | 174 F g^-1^ |  |
| NaCl/KCl-T |  |  | 155 F g^-1^ |  |
| LiCl/KCl-T |  |  | 138 F g^-1^ |  |
| LiCl/NaCl-T |  |  | 100 F g^-1^ |  |
| LiCl-T (templated) | 1M LiOH | 0.8 V  1 A g^-1^ | 189 F g^-1^ | ^[1]^ |
| NaCl-T (templated) | 1M NaOH |  | 204 F g^-1^ |  |
| KCl-T (templated) | 1M KOH |  | 205 F g^-1^ |  |
| RbCl-T (templated) | 1M RbOH |  | 203 F g^-1^ |  |
| CsCl-T (templated) | 1M CsOH |  | 222 F g^-1^ |  |
| RbCl-T (templated) | 0.5M Li_2_SO_4_ | 1.5 V  1 A g^-1^ | 127 F g^-1^ | ^[3]^ |
| CsCl-T (templated) |  |  | 147 F g^-1^ |  |
| BP2000 (Cabot, commercial) | 1M Li_2_SO_4_ | 1.6 V  1 A g^-1^ | 100 F g^-1^ | ^[27]^ |
| 507-20 (Kynol, commercial) | 40wt% KOAc | 1.8 V  1 A g^-1^ | 130 F g^-1^ | ^[28]^ |
| 5092-20 (Kynol, commercial) |  |  | 130 F g^-1^ |  |
| C-Temp (CsCl-templated) |  |  | 115 F g^-1^ |  |
| R3 Extra (Norit, commercial) |  |  | 80 F g^-1^ |  |
| MAC-2 (steam activated) |  |  | 110 F g^-1^ |  |
| YP50F (Kuraray, commercial) | Acetate buffer | 1.5 V  1 A g^-1^ | 91 F g^-1^ | ^[29]^ |
| Kynol 507-20 (Kynol, commercial) | 1M Li_2_SO_4_ | 1.6 V  1 A g^-1^ | 107 F g^-1^ | ^[30]^ |
| BP2000 (Cabot, commercial) |  |  | 95 F g^-1^ |  |


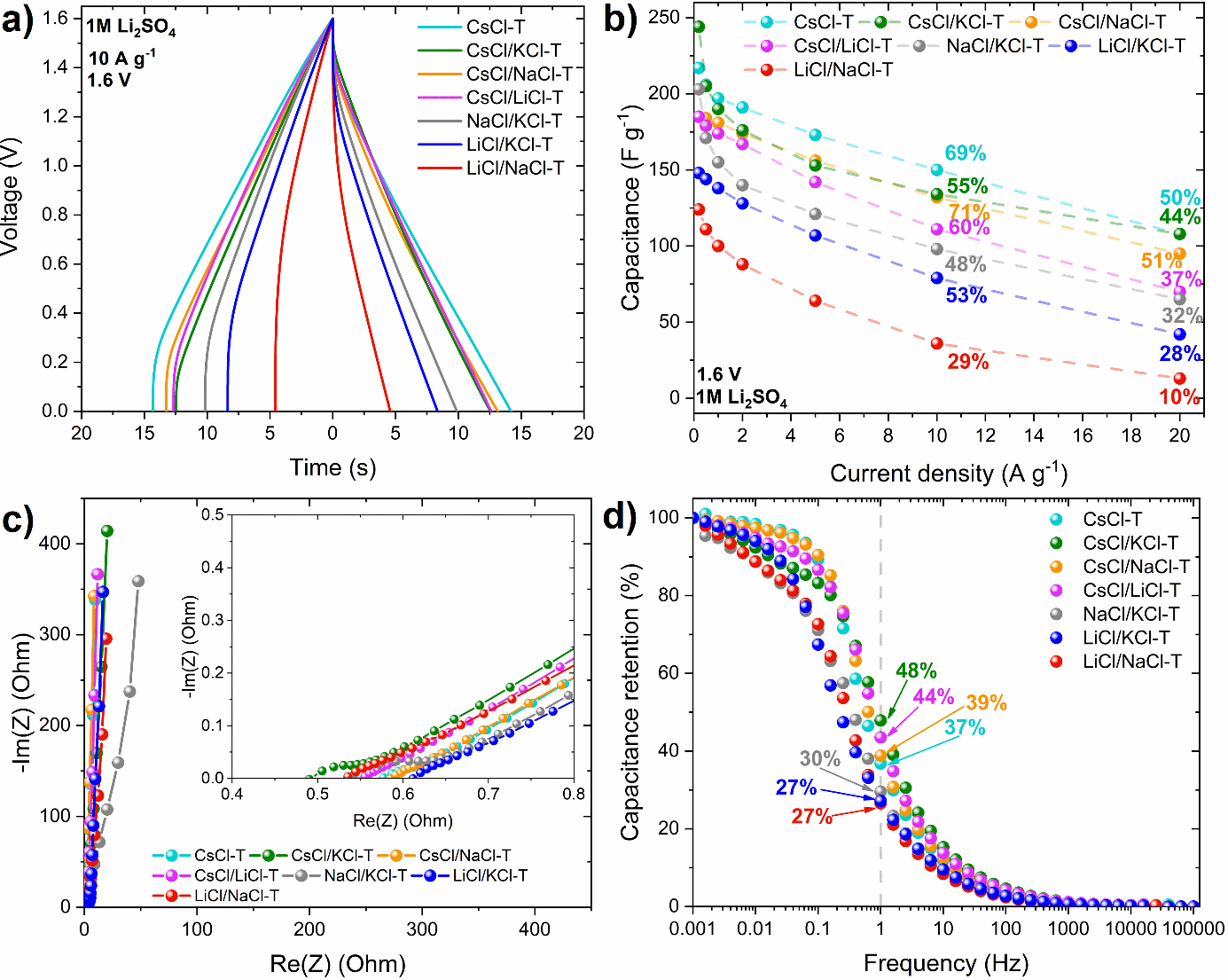


**Fig. S7** Electrochemical performance of salt templated carbons-based EDLCs operating in 1M Li_2_SO_4_ at 1.6 V: **a****)** galvanostatic charge/discharge at 10 A g^-1^, **b)** gravimetric capacitance (F g^-1^) *vs.* current density (A g^-1^), **c)** Nyquist plot (at 0 V), d) capacitance retention (%) *vs.* frequency (Hz).


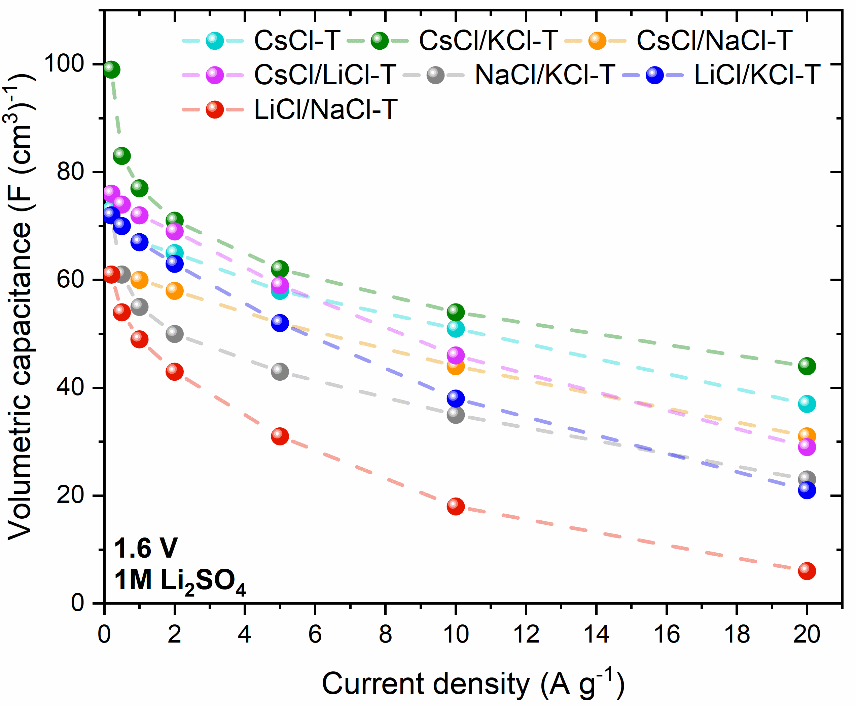


**Fig. S8** Volumetric capacitance (F cm^-3^) *vs.* current density (A g^-1^) of salt templated carbons-based EDLCs operating in 1M Li_2_SO_4_ up to 1.6 V.


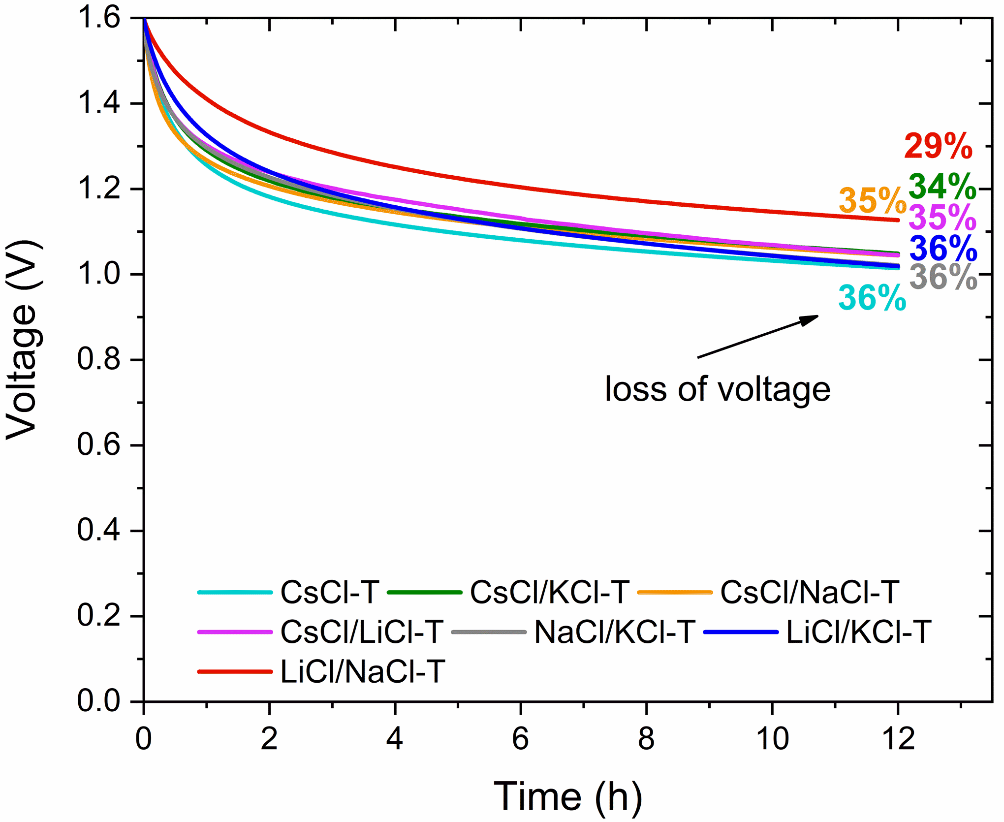


**Fig. S9** Self-discharge of salt templated carbons-based EDLCs operating in 1M Li_2_SO_4_.


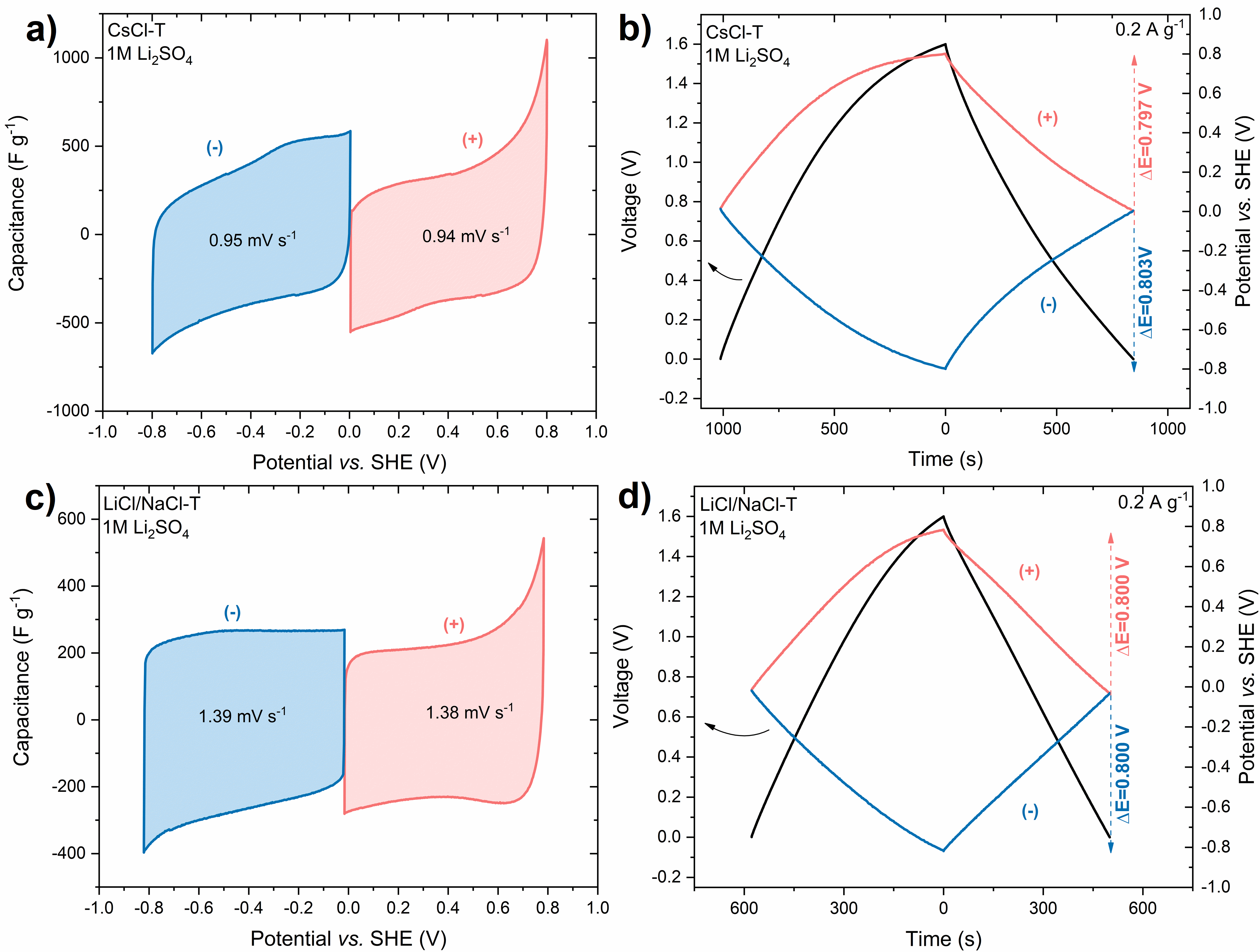


**Fig. S10** (left) Cyclic voltammetry curves in the potential ranges determined from 0.2 A g^-1^ galvanostatic charge/discharge recorded in 3-electrode set-up EDLC operating in 1M Li_2_SO_4_ at 1.6 V for **a)** CsCl-T, **c)** LiCl/NaCl-T salt templated carbons. (right) Galvanostatic charge/discharge profiles recorded in 3 electrode set-up at 0.2 A g^-1^ for EDLC operating in 1M Li_2_SO_4_ at 1.6 V for **b)** CsCl-T, **d)** LiCl/NaCl-T salt templated carbons.


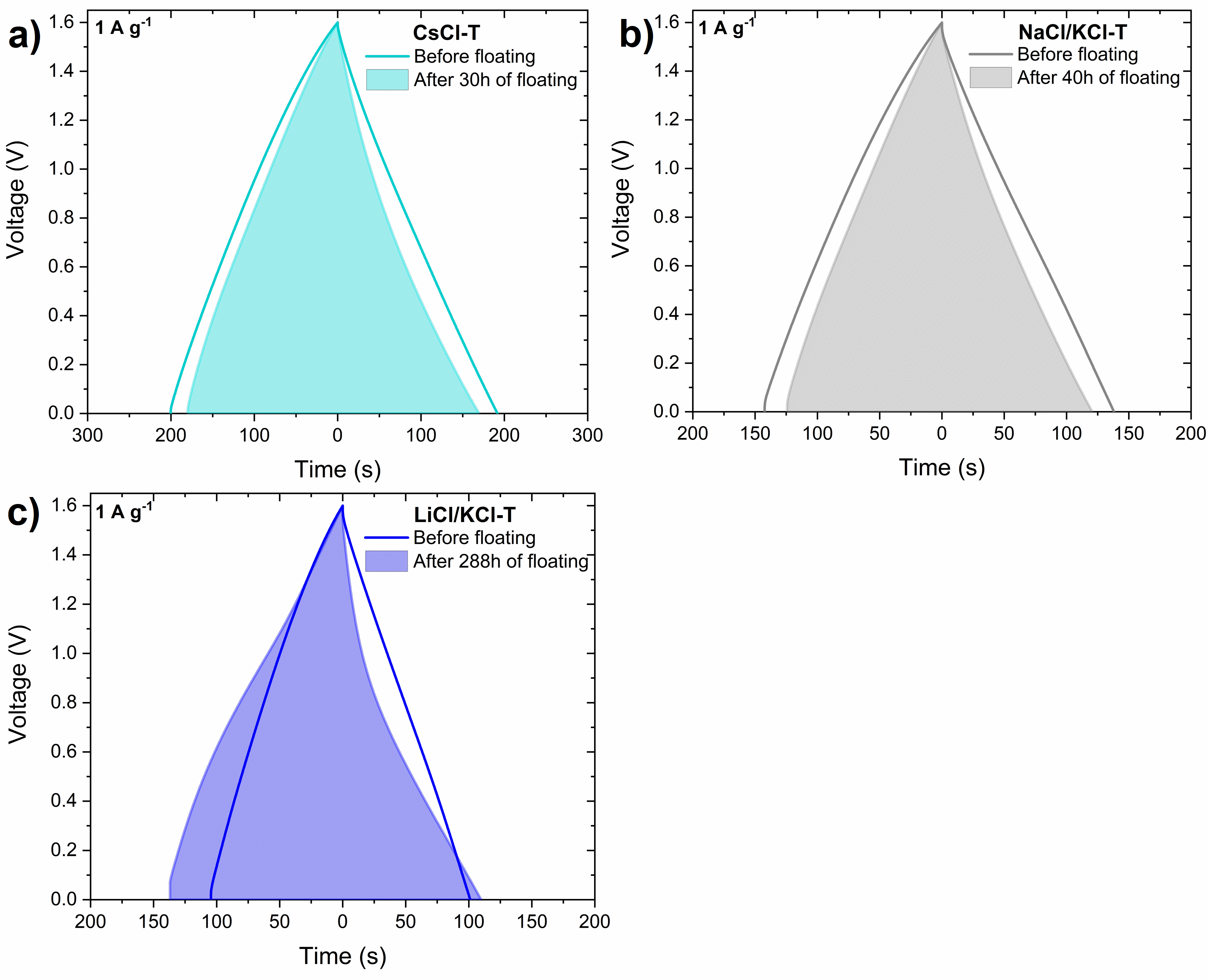


**Fig. S11** Galvanostatic charge/discharge before and after floating test of selected salt templated carbons-based EDLCs operating in 1M Li_2_SO_4_ at 1.6 V, 1 A g^-1^ **a)** CsCl-T, **b)** NaCl/KCl-T, **c)** LiCl/KCl-T.


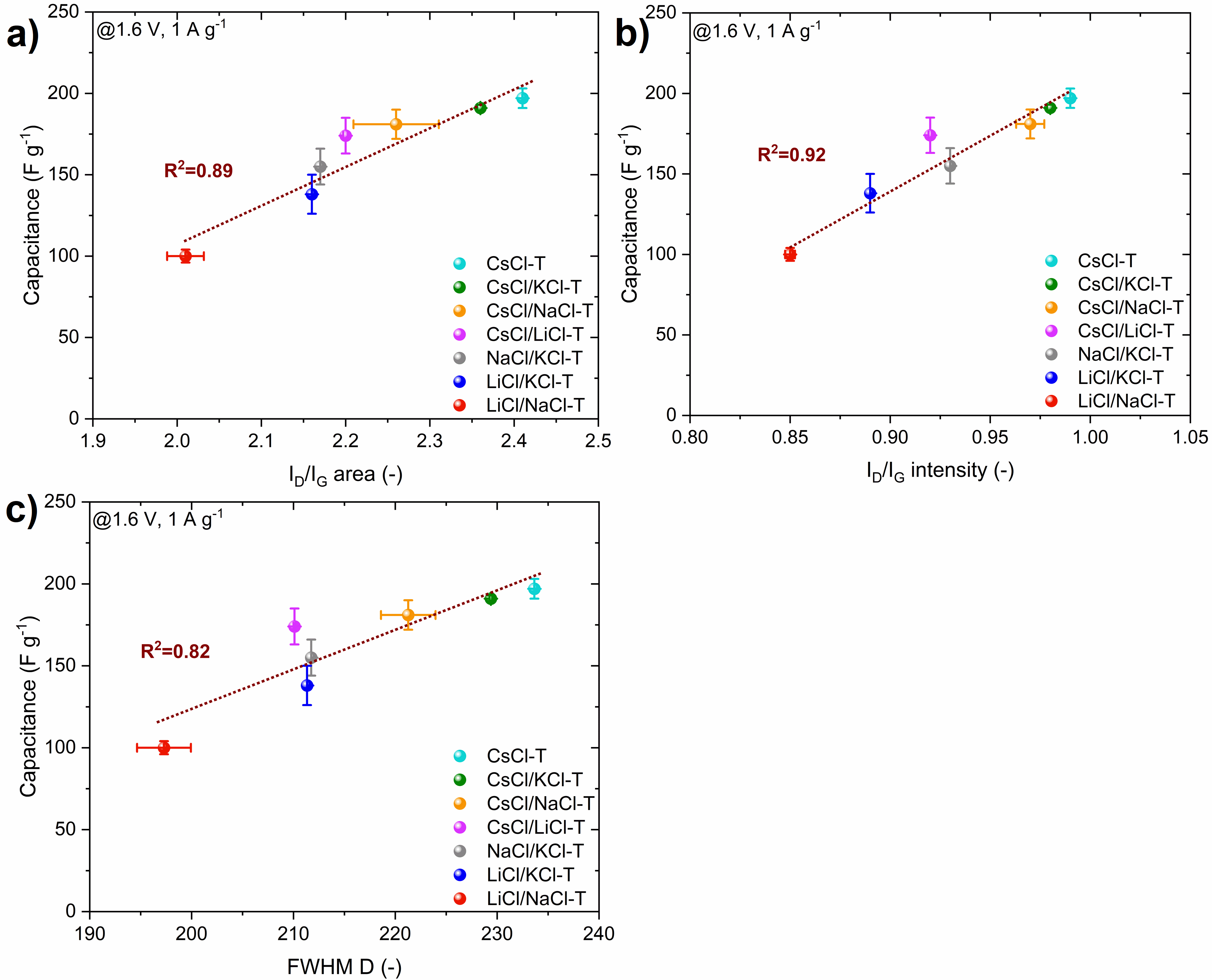


**Fig. S12** Correlation of gravimetric capacitance (1 A g^-1^, 1.6 V) and selected Raman spectral parameters of salt templated carbons after two peak fitting utilizing Lorentzian fitting function **a)** D peak area ratios related to the area of G band (I_D_/I_G_ area), **b)** D peak intensity ratios related to the intensity of G band (I_D_/I_G_ intensity), **c)** full width at half maximum of D band (FWHM D).


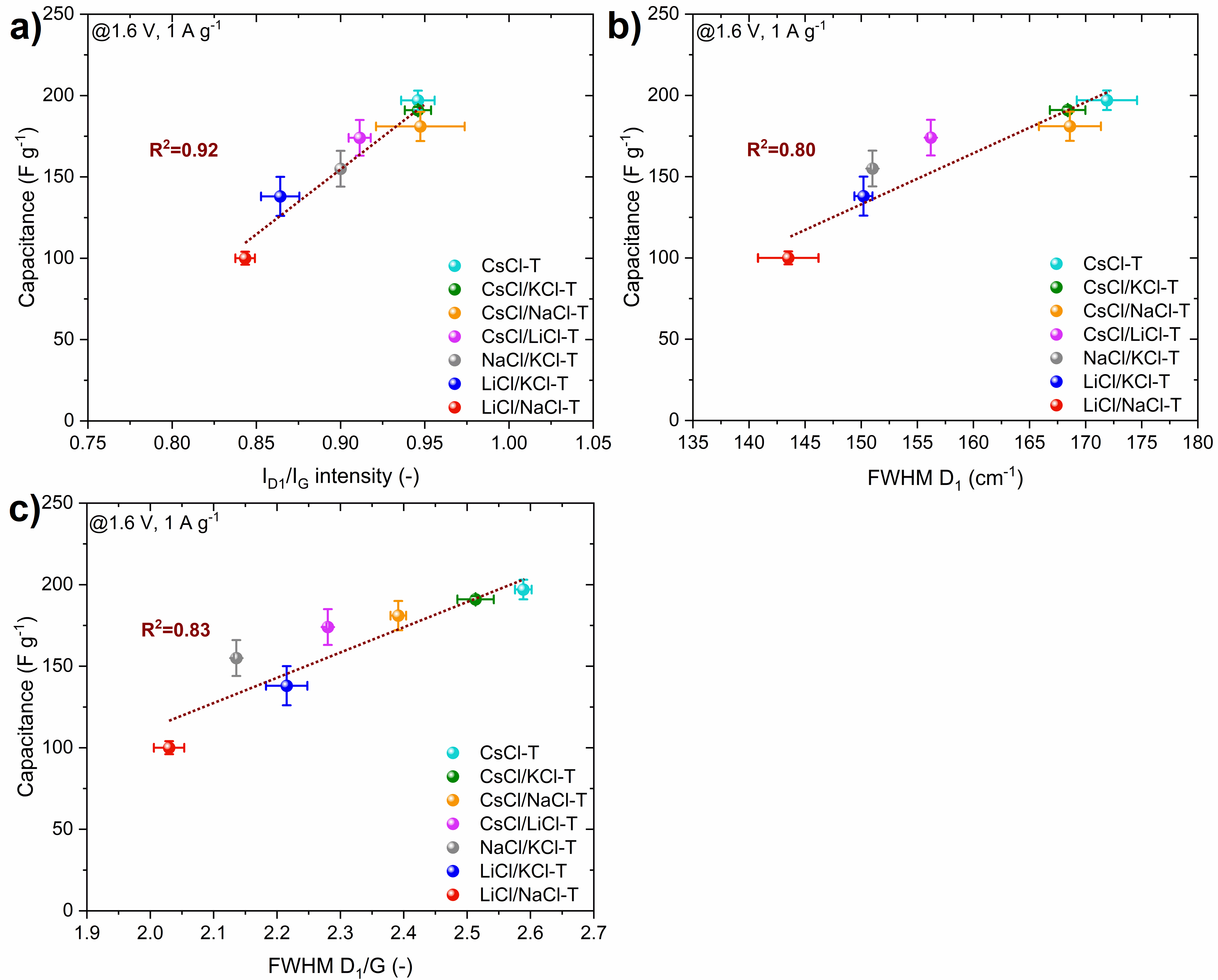


**Fig. S13** Correlation of gravimetric capacitance (1 A g^-1^, 1.6 V) and selected Raman spectral parameters of salt templated carbons after four peak fitting utilizing Lorentzian fitting function **a)** D_1_ peak intensity ratios related to the intensity of G band (I_D1_/I_G_ intensity), **b)** full width at half maximum of D_1_ band (FWHM D_1_), **c)** full width at half maximum of D_1_ band related to the area of G band (FWHM D_1_/G).

**Tab. S8** Summary of coefficient of determinations (R^2^) for capacitance at several current densities
(1.6 V, 0.2 – 20 A g^-1^) and selected spectral parameters after four peaks deconvolution i.e., peak areas ratios related to the area of G band (R^2^ I_D1_/I_G_ area, R^2^ I_D2_/I_G_ area, R^2^ I_D3_/I_G_ area), peak intensities ratios related to the intensity of G band (R^2^ I_D1_/I_G_ intensity), full width at half maximum related to the area of G band (R^2^ FWHM D_1_/G).

| Current density  (A g^-1^) | R^2^ I_D1_/I_G_  area | R^2^  I_D1_/I_G_  intensity | R^2^ FWHM  D_1_/G | R^2^ I_D2_/I_G_  area | R^2^  I_D3_/I_G_  area |
| --- | --- | --- | --- | --- | --- |
| 0.2 | 0.70 | 0.76 | 0.61 | 0.77 | 0.70 |
| 0.5 | 0.89 | 0.91 | 0.72 | 0.69 | 0.75 |
| 1 | 0.91 | 0.92 | 0.83 | 0.60 | 0.70 |
| 2 | 0.90 | 0.90 | 0.83 | 0.54 | 0.67 |
| 5 | 0.91 | 0.91 | 0.83 | 0.55 | 0.68 |
| 10 | 0.93 | 0.94 | 0.85 | 0.61 | 0.74 |
| 20 | 0.95 | 0.97 | 0.86 | 0.69 | 0.79 |


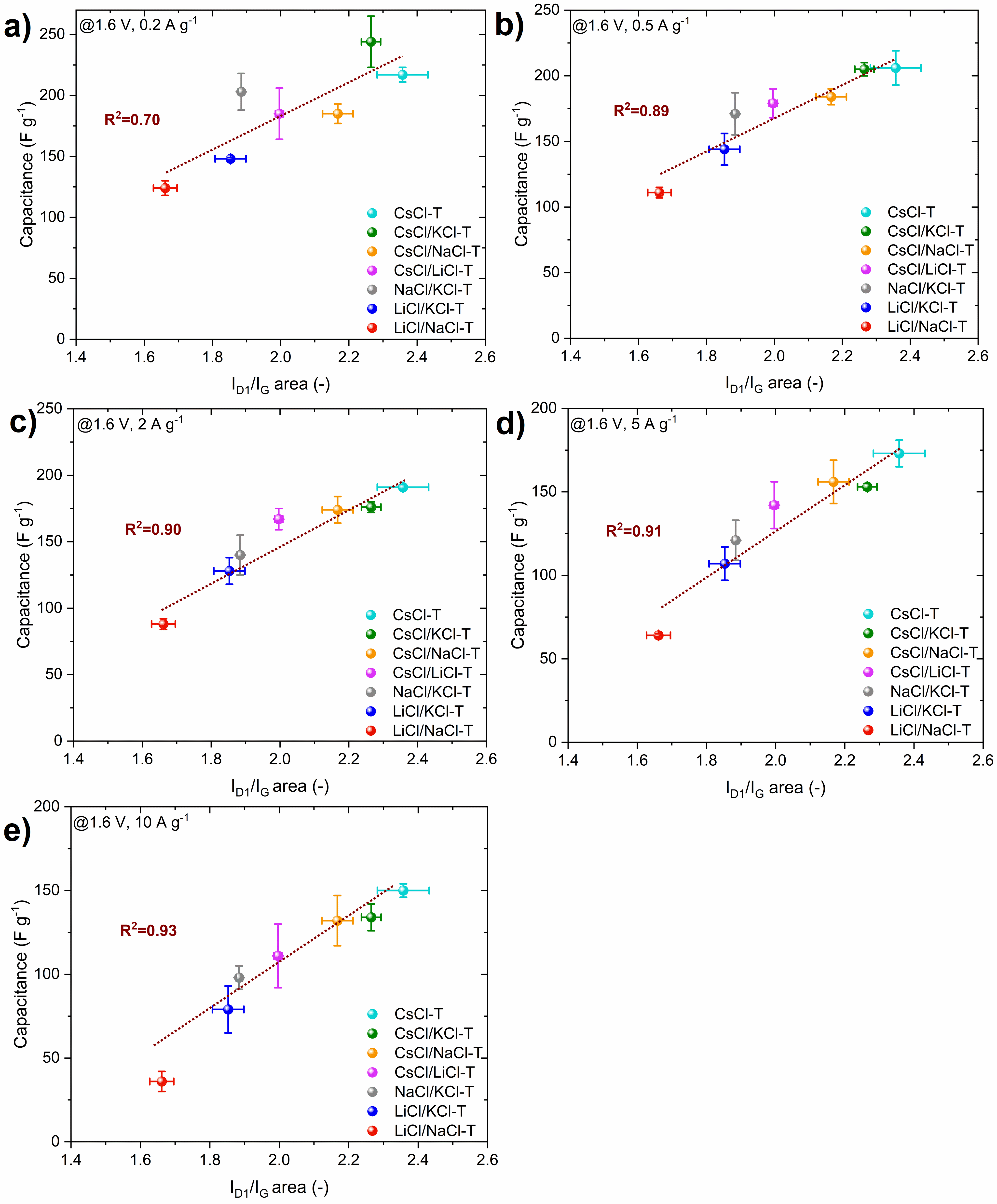


**Fig. S14** Correlation of gravimetric capacitance and I_D1_/I_G_ area ratio at different current densities **a)** 0.2 A g^-1^, **b)** 0.5 A g^-1^ **c)** 2 A g^-1^ **d)** 5 A g^-1^, **e)** 10 A g^-1^ of salt templated carbons.

**Tab. S9** Summary of the size (height) of stacked graphene layers (L_a_) and I_D_/I_G_ area ratio after two peaks fitting and of salt templated carbons.

| Material | L_a_  (nm) | I_D_/I_G_ area  (-) |
| --- | --- | --- |
| CsCl-T | 7.98 | 2.41 |
| CsCl/KCl-T | 8.15 | 2.36 |
| CsCl/NaCl-T | 8.51 | 2.26 |
| CsCl/LiCl-T | 8.74 | 2.20 |
| NaCl/KCl-T | 8.86 | 2.17 |
| LiCl/KCl-T | 8.90 | 2.16 |
| LiCl/NaCl-T | 9.56 | 2.01 |

**Tab. S10** Elemental analysis results, which were used to estimate the % of the oxygen in salt templated carbons.

| Material | Oxygen (wt %) |
| --- | --- |
| CsCl-T | 3.1 |
| CsCl/KCl-T | 3.4 |
| CsCl/NaCl-T | 3.5 |
| CsCl/LiCl-T | 2.1 |
| NaCl/KCl-T | 2.8 |
| LiCl/KCl-T | 2.1 |
| LiCl/NaCl-T | 2.4 |


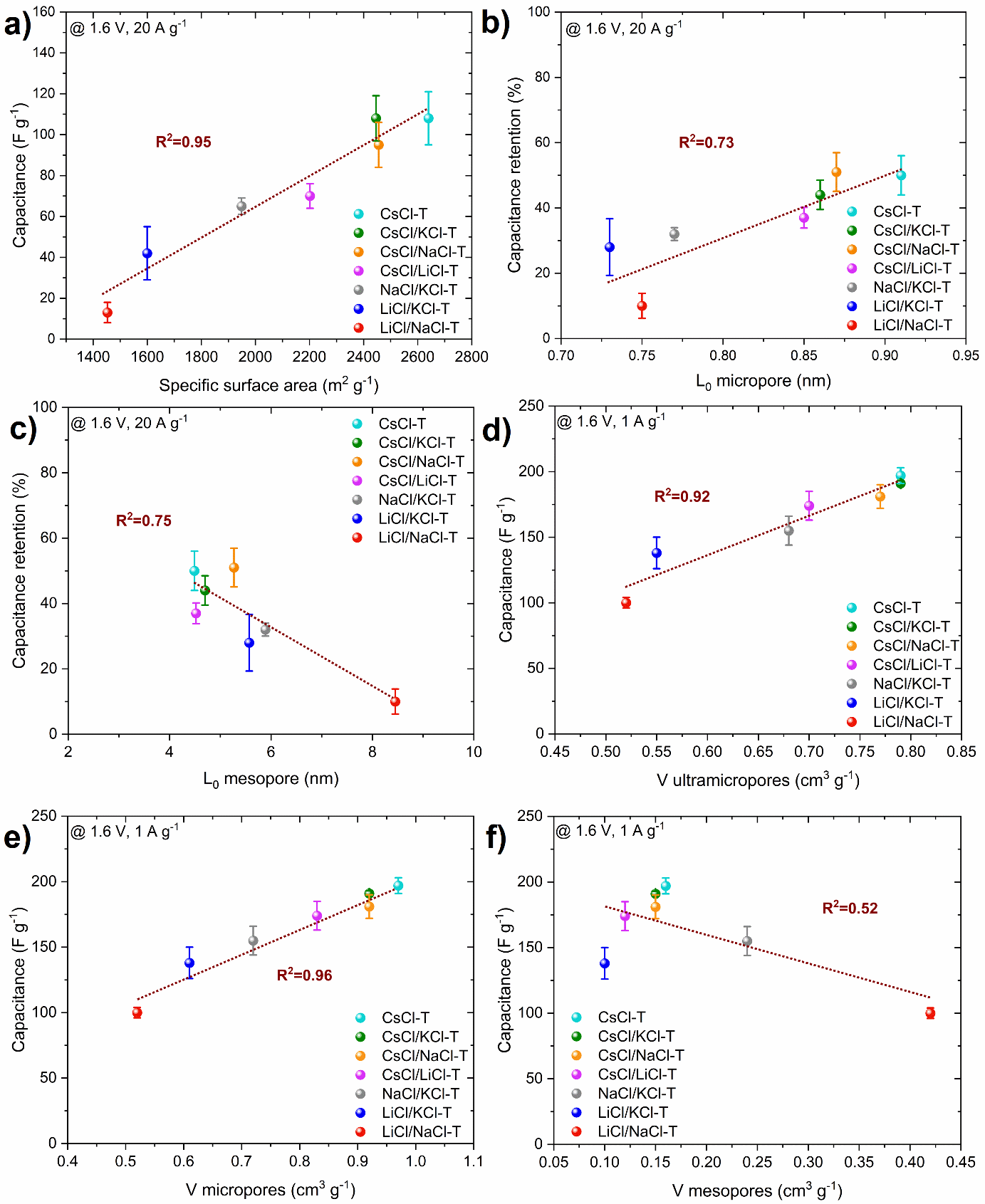


**Fig. S15 a)** Correlation of gravimetric capacitance (20 A g^-1^, 1.6 V) and **a)** specific surface area determined from nitrogen sorption at 77K of salt templated carbons. Correlation of capacitance retention (20 A g^-1^, 1.6 V) and **b)** the average diameter of micropore, **c)** the average diameter of mesopore of salt templated carbons determined from nitrogen sorption at 77K. Correlation of gravimetric capacitance (1 A g^-1^, 1.6 V) and **d)** volume of ultramicropores determined from carbon dioxide sorption at 273K, **e)** volume of micropores, and **f)** volume of mesopores determined from nitrogen sorption at 77K of salt templated carbons.


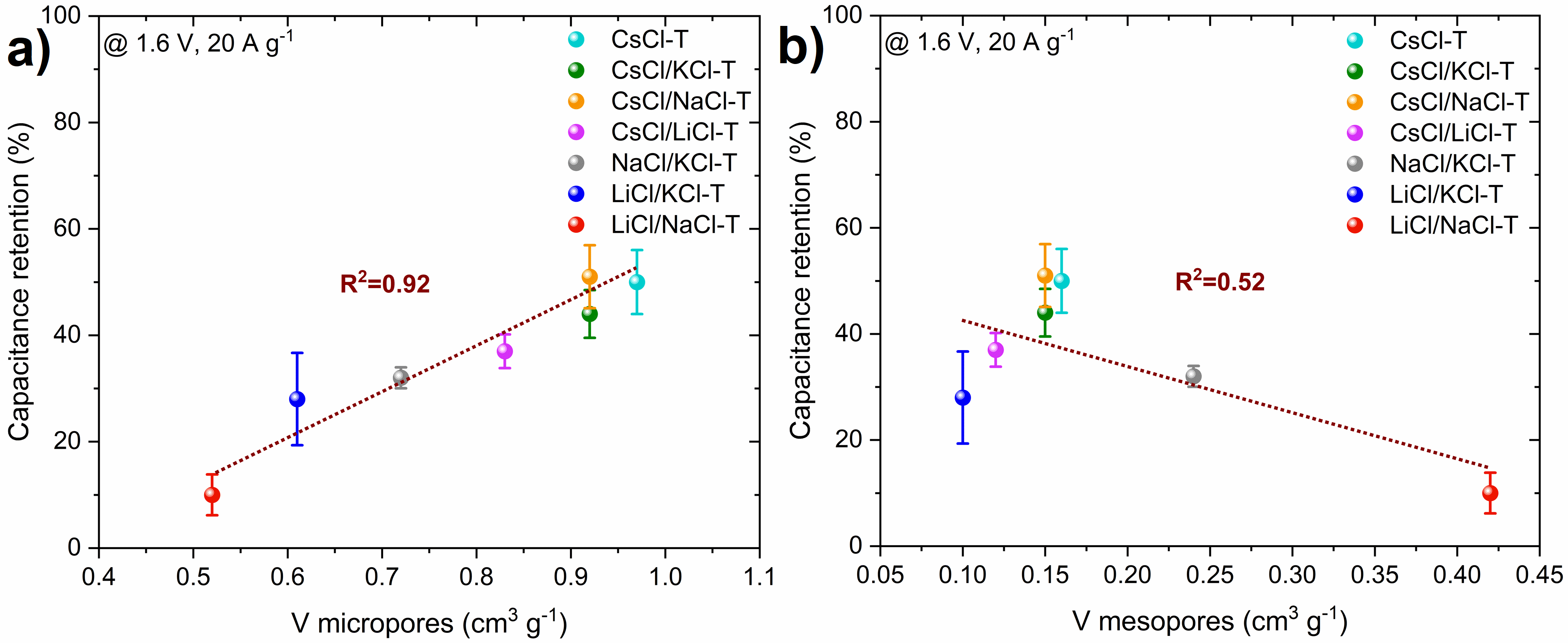


**Fig. S16** Correlation of capacitance retention (20 A g^-1^, 1.6 V) and **a)** volume of micropores, **b)** volume of mesopores of salt templated carbons determined from nitrogen sorption at 77K.

**References**

1 A. Platek-Mielczarek, C. Nita, C. Matei Ghimbeu, E. Frackowiak, K. Fic, Link between alkali metals in salt templates and in electrolytes for improved carbon-based electrochemical capacitors, *ACS Appl. Mater. Interfaces* **2021**, *13*, 2584.

2 C. Nita, M. Bensafia, C. Vaulot, L. Delmotte, C. Matei Ghimbeu, Insights on the synthesis mechanism of green phenolic resin derived porous carbons via a salt-soft templating approach, *Carbon* **2016**, *109*, 227.

3 A. Platek, C. Nita, C. Matei Ghimbeu, E. Frąckowiak, K. Fic, Electrochemical capacitors operating in aqueous electrolyte with volumetric characteristics improved by sustainable templating of electrode materials, *Electrochim. Acta* **2020**, *338*, 135788.

4 R. Kohli, in *Developments in Surface Contamination and Cleaning: Applications of Cleaning Techniques*, Elsevier, **2019**, pp. 619–680.

5 J. Sangster, A. D. Pelton, Phase diagrams and thermodynamic properties of the 70 binary alkali halide systems having common ions, *J. Phys. Ref. Data* **1987**, *16*, 509

6 I. V. Pentin, V. Saltykov, J. Nuss, J. C. Schön, M. Jansen, Theoretical and Experimental Exploration of the Energy Landscape of the Quasi‐Binary Cesium Chloride/Lithium Chloride System, *Chem. Euro. J.* **2012**, *18*, 3559.

7 A. L. Sihm, PhD thesis, Uppsala Universitet, **2019**.

8 A. Sadezky, H. Muckenhuber, H. Grothe, R. Niessner, U. Pöschl, Raman microspectroscopy of soot and related carbonaceous materials: Spectral analysis and structural information, *Carbon* **2005**, *43*, 1731.

9 S. Zallouz, T. Aurmond, A. Moissette, A. Sachse, C. Matei Ghimbeu, Zeolite-Templated Carbons as Supercapacitors: The Fundamental Role of Structural and Textural Properties, *ACS Appl. Energy Mater*. **2024**, *20*, 9142.

10 T. Ishii, T. Kyotani, in *Materials Science and Engineering of Carbon* (Eds.: M. Inagaki, F. Kang), Butterworth-Heinemann, **2016**, pp. 287–305.

11 J. L. Figueiredo, M. F. R. Pereira, M. M. A. Freitas, J. J. M. Órfão, Modification of the surface chemistry of activated carbons, *Carbon* **1999**, *37*, 1379.

12 R. P. Rocha, M. F. R. Pereira, J. L. Figueiredo, Characterisation of the surface chemistry of carbon materials by temperature-programmed desorption: An assessment, *Catal. Today* **2023**, *418*, 114136.

13 B. Réty, H.Y. Yiin, C. Matei Ghimbeu, Quantification of activated carbon functional groups and active surface area by TPD-MS and their impact on supercapacitor performance, *Energy Storage Mater.* **2025**, *74*, 103963.

14 N. R. Laine, F. J. Vastola, P. L. Jr. Walker, The importance of active surface area in the carbon-oxygen reaction, *J. Phys. Chem.* **1963**, *67*, 2030.

15 A. Beda, C. Vaulot, F. Rabuel, M. Morcrette, C. Matei Ghimbeu, The role of specific and active surface areas in optimizing hard carbon irreversible capacity loss in sodium ion batteries, *Energy Adv.* **2022**, *1*, 185.

16 S. Azmi, M. F. Koudahi, E. Frackowiak, Reline deep eutectic solvent as a green electrolyte for electrochemical energy storage applications, *Energy Environ. Sci.* **2022**, *15*, 1156.

17 J. W. Gittins, Y. Chen, S. Arnold, V. Augustyn, A. Balducci, T. Brousse, E. Frackowiak, P. Gómez-Romero, A. Kanwade, L. Köps, P. K. Jha, D. Lyu, M. Meo, D. Pandey, L. Pang, V. Presser, M. Rapisarda, D. Rueda-García, S. Saeed, P. M. Shirage, A. Ślesiński, F. Soavi, J. Thomas, M.M. Titirici, H. Wang, Z. Xu, A. Yu, M. Zhang, A. C. Forse, Interlaboratory study assessing the analysis of supercapacitor electrochemistry data, *J. of Power Sources* **2023**, *585*, 233637.

18 X. Liu, D. Lyu, C. Merlet, M. J. A. Leesmith, X. Hua, Z. Xu, C. P. Grey, A. C. Forse, Structural disorder determines capacitance in nanoporous carbons, *Science* **2024**, *384*, 321.

19 E. Pamete, L. Kops, F.A. Kreth, S. Pohlmann, A. Varzi, T. Brousse, A. Balducci, V. Presser, The many deaths of supercapacitors: degradation, aging, and performance fading, *Adv. Energy Mater.* **2023**, *28*, 2301008.

20 R.D. Shannon, R. D. Prewitt, C. T. Effective Ionic Radii in Oxides and Fluorides. *Acta Cryst B* **1969**, *25*, 925.

21 F. David, V. Vokhmin, G. Ionova, Water Characteristics Depend on the Ionic Environment. Thermodynamics and Modelisation of the Aquo Ions. *J. of Mol. Liq.* **2001**, *90*, 45.

22 A.G. Volkov, S. Paula, D.W. Deamer, Two Mechanisms of Permeation of Small Neutral Molecules and Hydrated Ions across Phospholipid Bilayers. *Bioelectrochemistry and Bioenergetics* **1997**, *42*, 153.

23 E.R. Nightingale Jr., Phenomenological Theory of Ion Solvation. Effective Radii of Hydrated Ions*, J. Phys. Chem.* **1959***, 63,* 1381.

24 D. W. Smith, Ionic hydration enthalpies, J. Chem. Educ. **1977**, *54*, 540.

25 M.Y. Kiriukhin, K.D. Collins, Hydration Numbers for Biologically Important Ions. *Biophysical Chemistry* **2002**, *99*, 155.

26 F. Thaunay, A.A. Hassan, R.J. Cooper, E.R. Williams, C. Clavaguéra, G. Ohanessian, Hydration of the Sulfate Dianion in Size-Selected Water Clusters: From SO42−(H2O)9 to SO42−(H2O)13. *Int. J. Mass Spectrom.* **2017**, *418*, 15.

27 S. Azmi, A. Klimek, E. Frackowiak, Electrochemical capacitor in aqueous electrolyte with long lifespan improved by hydrogen bond donor addition, *Materials Today* **2023**, *68*, 34.

28 S. Zallouz, L. Dentzer, C. Matei Ghimbeu, Carbon Capacitors Operating at 1.8 V Based on Concentrated Aqueous Electrolytes: Impacts of the Electrolyte Concentration and Carbon Properties. *ACS Appl. Energy Mater.* **2024**, *7*, 1448.

29 A. Klimek, M. Tobis, E. Frackowiak, Effect of a buffer/iodide electrolyte on the performance of electrochemical capacitors, *Green Chem.* **2024**, *26*, 6684.

30 A. Platek-Mielczarek, J. Piwek, E. Frackowiak, K. Fic, Ambiguous Role of Cations in the Long-Term Performance of Electrochemical Capacitors with Aqueous Electrolytes. *ACS Appl. Mater. Interfaces* **2023**, *15*, 23860.
